# Fully automated leg tracking of *Drosophila* neurodegeneration models reveals distinct conserved movement signatures

**DOI:** 10.1371/journal.pbio.3000346

**Published:** 2019-06-27

**Authors:** Shuang Wu, Kah Junn Tan, Lakshmi Narasimhan Govindarajan, James Charles Stewart, Lin Gu, Joses Wei Hao Ho, Malvika Katarya, Boon Hui Wong, Eng-King Tan, Daiqin Li, Adam Claridge-Chang, Camilo Libedinsky, Li Cheng, Sherry Shiying Aw

**Affiliations:** 1 Bioinformatics Institute, Agency for Science, Technology and Research, Singapore; 2 Institute of Molecular and Cell Biology, Agency for Science, Technology and Research, Singapore; 3 Duke-NUS Graduate Medical School, Neuroscience and Behavioural Disorders, Singapore; 4 National University of Singapore, Department of Biological Sciences, Singapore; 5 National Neuroscience Institute, Singapore General Hospital, Singapore; 6 Singapore Institute for Neurotechnology (SiNAPSE), Singapore; 7 National University of Singapore, Department of Psychology, Singapore; 8 Department of Electrical and Computer Engineering, University of Alberta, Edmonton, Alberta, Canada; University of Oxford, UNITED KINGDOM

## Abstract

Some neurodegenerative diseases, like Parkinsons Disease (PD) and Spinocerebellar ataxia 3 (SCA3), are associated with distinct, altered gait and tremor movements that are reflective of the underlying disease etiology. *Drosophila melanogaster* models of neurodegeneration have illuminated our understanding of the molecular mechanisms of disease. However, it is unknown whether specific gait and tremor dysfunctions also occur in fly disease mutants. To answer this question, we developed a machine-learning image-analysis program, Feature Learning-based LImb segmentation and Tracking (FLLIT), that automatically tracks leg claw positions of freely moving flies recorded on high-speed video, producing a series of gait measurements. Notably, unlike other machine-learning methods, FLLIT generates its own training sets and does not require user-annotated images for learning. Using FLLIT, we carried out high-throughput and high-resolution analysis of gait and tremor features in *Drosophila* neurodegeneration mutants for the first time. We found that fly models of PD and SCA3 exhibited markedly different walking gait and tremor signatures, which recapitulated characteristics of the respective human diseases. Selective expression of mutant SCA3 in dopaminergic neurons led to a gait signature that more closely resembled those of PD flies. This suggests that the behavioral phenotype depends on the neurons affected rather than the specific nature of the mutation. Different mutations produced tremors in distinct leg pairs, indicating that different motor circuits were affected. Using this approach, fly models can be used to dissect the neurogenetic mechanisms that underlie movement disorders.

## Abstract 2

This study uses automated leg tracking to characterise gait and tremor features in fruit fly models of Parkinson’s disease and spinocerebellar ataxia 3, finding movement features that resemble characteristics of the respective human diseases.

## Introduction

Walking requires coordination of the central and peripheral nervous systems and the musculoskeletal system. Hence, neurological and musculoskeletal pathologies can manifest as stereotypic movement abnormalities. For example, patients with Parkinsons disease (PD) exhibit slowed movements (bradykinesia), rigidity, and resting tremor [1], while patients with cerebellar ataxias, e.g., Spinocerebellar ataxia Type 3 (SCA3), exhibit stumbling, jerky, uncoordinated movements, and action tremor [2–5]. One common disease signature is tremor: uncontrolled shaking of the body or appendages. While tremors can be temporarily triggered by physiological states like stress and anger, pathological tremors are often symptomatic of an underlying neurological disorder. There is no cure for tremor, and its pathophysiological causes are not understood [6,7]. Movement disorders exhibit significant phenotypic heterogeneity [3,8–10]. Detailed molecular and physiological characterization of associated neuronal circuits, and experiments to link gene function to neuronal activity and specific behavioral outputs can help us to understand how fundamental mechanisms of motor control are disrupted in disease. Our understanding of movement disorders can benefit greatly from study in a genetic animal model with a relatively small and manipulatable nervous system.

Despite the differences in anatomy and scale between human and fly brains, *Drosophila* disease models have made substantial contributions to our understanding of the mechanisms underlying human neurodegenerative diseases [11,12]. However, while fly disease models recapitulate molecular features of disease, and fly and mammalian neurons share similar molecular machinery [13], it is unknown whether flies can be used to understand how aging and disease affect motor control of gait, as gait and tremor characteristics of fly neurodegeneration models have not been previously quantified due to technical challenges. Quantitative measurement of locomotion in neurodegeneration fly mutants has been confined to more general assessments, e.g., the climbing assay [14]. Tremor behavior, in particular, has eluded characterization; while flies of several mutant genotypes have been reported to show tremor [15–18], the quantitative characteristics of these movements have not been determined.

To characterize movements as rapid and fine as tremors in the fly requires an accurate, automated method for leg tracking. While gait has been studied in flies, until recently, previous studies employed either foot-printing–based approaches that report only contact points with a detection surface, limiting tracking output [19,20], leg-marking–based techniques that track distinct marked spots on the legs, requiring laborious marking of each leg [21], or semi-automated algorithms that require a considerable degree of user annotation and/or user-led optimization for accuracy [22–24], which are hence laborious and time-consuming. Therefore, these methods were not feasible for use on the large volume of data required to quantify rapid and fine tremors in suspended legs. Two recent studies describe deep learning approaches for marker-less tracking [25,26], which could also be applied to study gait [26]. These methods are not yet fully automated as they require a substantial number of user-annotated training images; however, they are highly versatile, as they can be applied to various models and behaviors.

To enable accurate and automated leg tracking, we developed Feature Learning-based LImb segmentation and Tracking (FLLIT), a machine learning method able to automatically track leg movements of freely moving flies from high-speed video, with high accuracy and minimal user input. FLLIT then automatically produces a series of calculated gait parameters. Of note, FLLIT generates its own training sets and does not require user annotation of images for training. Hence, it is less laborious than other machine learning approaches, which generally require hundreds to thousands of hand-annotated training images for learning. Using FLLIT to characterize gait in fly models for PD and SCA3, we found that these mutants exhibited distinct movement signatures that recapitulated aspects of the movement dysfunctions in human patients. Gait and tremor analyses in fly models can enable future studies into the genetic and neural basis underlying subtle movement dysfunctions like tremors.

## Results

### System setup and computational workflow

**Fig 1:**
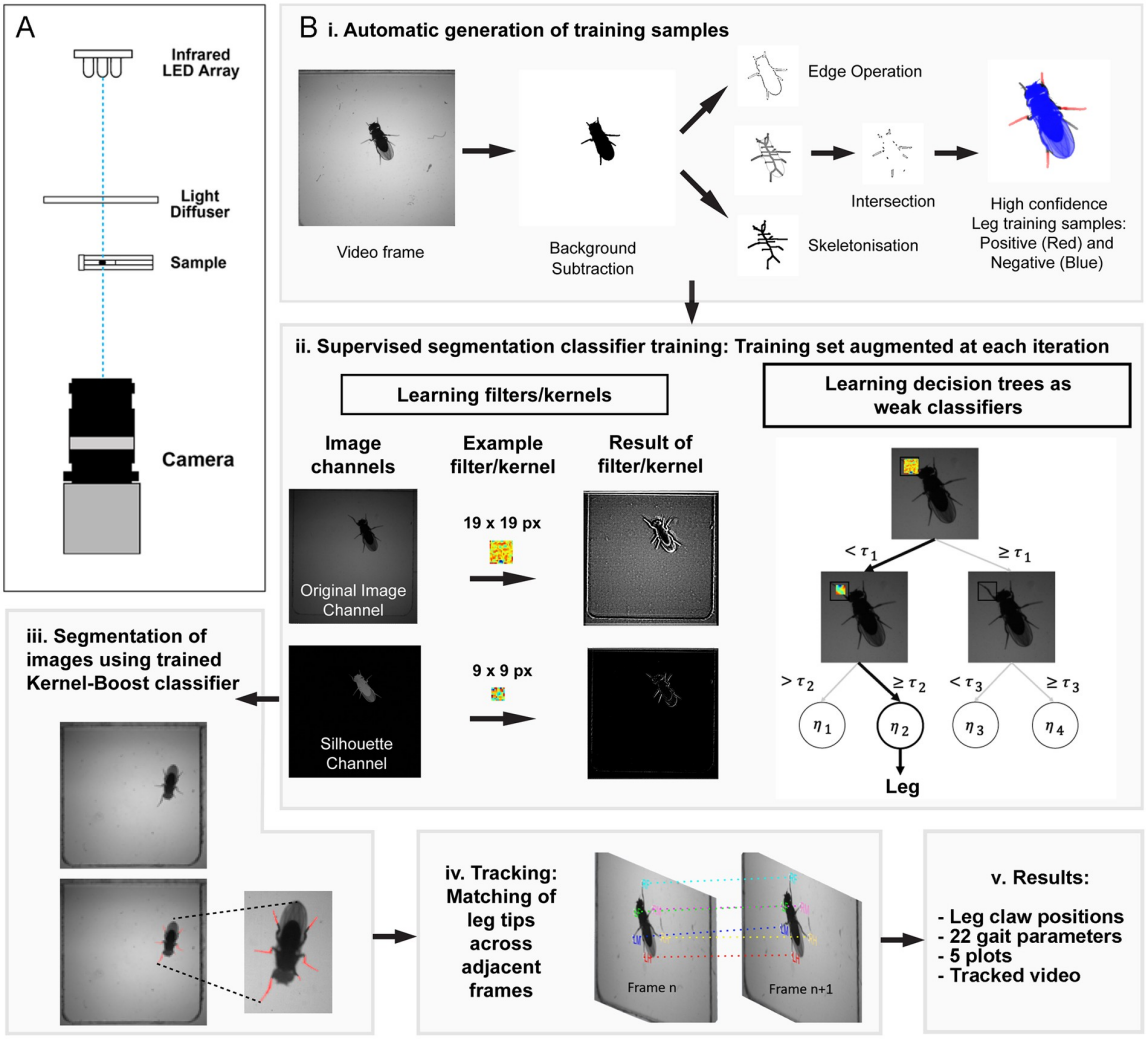
FLLIT system setup and overview of computational workflow. A. Camera and arena setup used for video capture. B. Segmentation and tracking procedure. i) Training samples are automatically generated, without any user input, by identifying high-confidence leg px (shown in red; located at the intersection between skeletonization and edge morphological operations) and high-confidence non-leg pixels (shown in blue) (see text for details). ii) Training sets are learned and grown by iterative supervised segmentation to derive a classifier. iii) Segmentation of novel images is carried out using the trained classifier. iv) Tracking occurs by matching leg claw positions across adjacent frames. v) Results are given as positions of leg claws in each frame. FLLIT, Feature Learning-based Limb segmentation and Tracking; px, pixels.

We utilized a video recording setup that consists of a high-speed camera mounted below the sample, backlit by an infrared LED array (Fig 1A and S1 Fig). The computational workflow carried out by FLLIT consists of (i) automated training set generation, (ii) supervised learning of leg classification, (iii) application of the trained classifier to novel images for leg segmentation, (iv) leg tracking across frames, and (v) results output (Fig 1B). A brief overview of each stage of the workflow is detailed below.

#### Generating the training set

To identify and segment legs in each video frame, we use an iterative learning approach that incorporates steps for the automated preparation of training sets. Therefore, no user input is required for training. This sets FLLIT apart from other supervised machine-learning methods for tracking [25,26], which require tedious manual preparation of training samples. To achieve this, FLLIT automatically performs a series of image processing steps (Fig 1Bi) on a subset of the images from the video of interest: background subtraction [27], medial axis skeletonization, and edge extraction, to identify only high-confidence positive (leg; shown in red in Fig 1Bi) and negative (non-leg; shown in blue in Fig 1Bi) pixel examples for learning. (The grey regions, while part of the region of interest obtained after background subtraction, are not of high enough confidence to be determined as being either leg or non-leg and are hence not used for learning).

#### Identification of legs

FLLIT aims to learn a precise binary leg segmentation model from pixels in the training set using an iterative learning approach based on the Kernel–Boost method [28,29] (Fig 1Bii). Briefly, 30,000 positive and negative pixels are sampled from the high-confidence pool (e.g., from Fig 1Bi). Training examples are extracted as 41 x 41 square pixel image patches, consisting of a central positive/negative pixel of interest and its surrounding pixels. A boosting approach is then adopted in which we iteratively learn an ensemble of 100 weak learners that are combined to provide the eventual classifier. At each iteration, a set of 100 candidate convolution kernels (whose sizes vary from 4 x 4 to 19 x 19 square pixels) is randomly generated to extract features from the training patches. Employing different sized convolution kernels allows exploration of neighbouring pixels of an image patch at different scales to provide diverse receptive fields. Extracted features are then used to construct a decision tree of depth 5 (Fig 1Bii). A weak learner comprises the decision tree parameters as well as the associated convolution kernels. High-confidence pixel classification predictions at the current iteration are added to the training set of the next iteration, thus iteratively augmenting the training set in favor of the safe and/or high-confidence predictions. The feedback and retraining loops expand the training set to build a stronger segmentation model at each iteration. The hyperparameters used are shown in Table 3.

After the classifier has been trained, it can be used to predict leg pixels in novel images (based on a set classification threshold) (Fig 1Biii), and the predicted leg pixels then grouped as legs. These segmented leg images are saved by FLLIT and can be used for further analyses.

#### Leg tracking

After the legs are identified, tracking is performed (Fig 1Biv and S1 Video). Each frame is body centered, and leg claw positions are matched across adjacent frames using the Hungarian method [30]. The tracked leg-claw data for each leg can then be extracted as a comma separated value(s) (CSV) file for analysis (Fig 1Bv).

#### FLLIT analysis speed and software

We analysed the time taken by FLLIT for data analysis. For a video of 1,000 frames, automated generation of training sets, learning and segmentation requires about 20 min in total. Automated tracking takes about 5 min; manual corrections takes about 1–2 s per correction, while analysis of gait parameters takes about 5 min (tested on an processor of 2.50 GHz with a RAM of 8 GB).

The FLLIT software provides an interface for automated tracking of leg movements from high-speed videos. Besides the set of data files of tracked body and leg positions, FLLIT also automatically calculates 20 body and gait parameters and provides 5 plots and a video for visualizing the tracked data (Table 1). The FLLIT program, readme and sample data can be downloaded from https://github.com/BII-wushuang/FLLIT.

**Table 1:** Movement and gait data automatically computed by FLLIT include raw body and leg claw position data, as well as 20 leg movement parameters, 5 plots, and a tracked video.

FLLIT Parameters			File
**Raw data**	Body position	Positional coordinates of the body centroid in each frame	CoM.csv
	Body trajectory	Angle of rotation of the body axis in degrees (relative to the arena y-axis)	CoM.csv
	Arena-centered leg claw positions	Positional coordinates of each leg claw in each frame based on arena coordinates	trajectory.csv
	Body-centered leg claw positions	Positional coordinates of each leg claw in each frame with reference to the body centroid	norm_trajectory.csv
**Body parameters**	Body length (mm)	Length of the sample animal estimated in each frame of the video (from the anterior-most position on the head to the posterior-most position on the wings)	bodylength.csv
	Instantaneous body velocity (mm/s)	Instantaneous velocity of the body (centroid) in the sample animal	bodyvelocity.csv
**Stride parameters (for each stride of each leg)**	Stride duration (ms)	The duration of a stride event	StrideParameters.csv
	Stride period (ms)	The duration from one stride event to the next	
	Stride displacement/length (mm)	The displacement distance of the leg claw during a stride event	
	Stride path covered (mm)	The total path covered by the leg claw during a stride event	
	Take-off position (posterior extreme position)^#^ (mm)	The posterior extreme position of a leg claw at the start of a stride event, relative to the body centroid (given in x and y mm coordinates)	
	Landing position (Anterior extreme position)^#^ (mm)	The anterior extreme position of a leg claw at the end of a stride event, relative to the body centroid (given in x and y mm coordinates)	
	Stride amplitude (mm)	The displacement along the direction of motion for a stride event	
	Stance linearity^#^ (mm)	The deviation of the stride path from a curve smoothed over (at 20-ms intervals) the corresponding anterior and posterior extreme positions of the stride.	
	Stride stretch (mm)	The distance of the leg tip position from the body center in the middle of a stride event	
**Leg parameters(for each leg)**	Leg speed (mm/s)	The instantaneous speed of each leg	LegParameters.csv
	Gait index ^#^	A gait index of 1 corresponds to a tripod gait, −1 corresponds to a tetrapod gait while 0 constitutes an noncanonical gait. In our implementation, the gait index is obtained by a moving average over a 120 ms window.	
	Movement percentage (%)	Percentage of the time that a leg is in motion	
	Mean stride period (ms)	Average duration from one stride event to the next	
	Footprint regularity^#^ (mm)	Average standard deviation of the posterior and anterior extreme positions of a leg	
	Leg trajectory domain area (mm^2^)	The area of the minimal convex hull that contains the entire leg trajectory in the body-centered frame of reference	
	Leg trajectory domain length and width (mm)	Obtained via the maximum projected distance of the claw positions onto the major (for domain length) and minor (for domain width) principal axes	
	Leg trajectory domain intersection (mm^2^)	The intersection/overlap area between each possible pair of leg domains	LegDomainOverlap.csv
	Stance width (mm)	Average of the distance between the AEP and PEP of the left and right middle legs	StanceWidth.csv
**Plots and Video generated**	Body trajectory and turning points	Plot of overall body trajectory and identification of positions where turns >50 deg occurred between two neighboring linear segments of a simplified trajectory drawn using the Douglas–Peucker algorithm	BodyTrajectory.pdf
	Body velocity	Plot of instantaneous velocity of the body (centroid) in the sample animal (see above)	BodyVelocity.pdf
	Leg claw positions and trajectories in body-centered frame of reference	Traces of the paths taken by each leg throughout the whole video	LegDomain.pdf
	Gait plots, change to leg speed	Plot of instantaneous speed of each leg (see above)	Gait.pdf
	Gait index	Plot of gait index over time (see above)	GaitIndex.pdf
	Video of tracked data in arena- and body-centered views	Video showing the fly and individual tracked legs in the arena- and body-centered views, with a plot of the instantaneous body trajectory and the corresponding instantaneous lateral (x) and vertical (y) positions of each leg marked in different colors	Video.mp4

**Abbreviations**: AEP, anterior extreme position; CoM, center of mass; PEP, posterior extreme position.

^#^ From reference [19].

### Accuracy of FLLIT segmentation and tracking results

**Fig 2:**
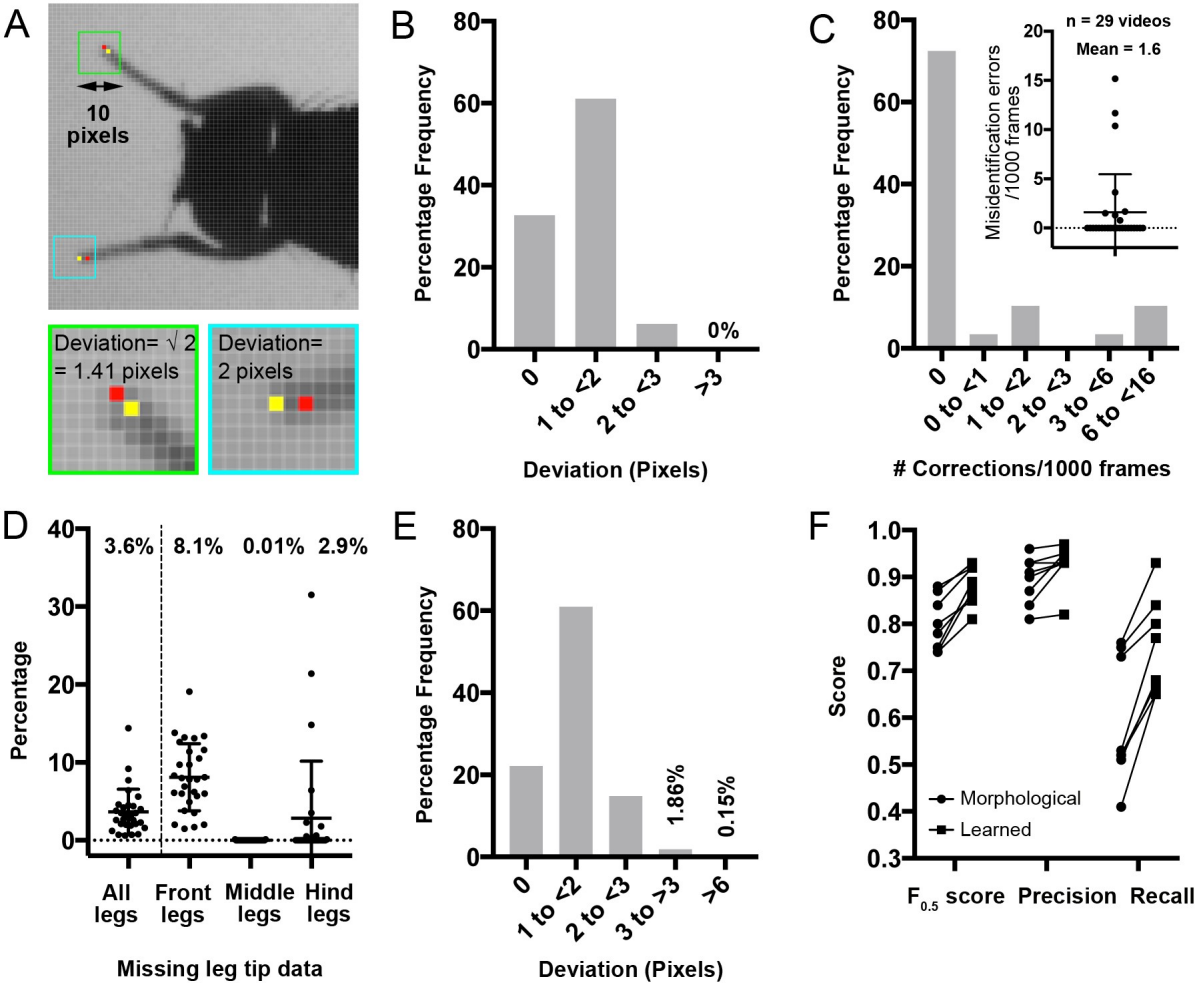
Accuracy of FLLIT segmentation and tracking results. A. Representative images of wild-type *Drosophila* legs taken using the default settings and the manual leg-tip positions identified by two different human users. Blue and green insets are 10 pixels wide and show the respective boxed regions in the top image. Red and yellow dots represent the pixels identified as tip pixels by the two users, within the respective blue and green boxes. B. Frequency distribution of the deviation (in pixels) between leg-tip positions annotated by the two users (*n* = 54 frames, 324 leg tips, from two videos). Discrepancies can occur in both the x and y directions and are represented as the Euclidean distance between the two pixels. C. Number of corrections required for misidentified legs, normalised to per 1,000 frames (mean = 1.7 corrections; *n* = 29 videos, 15,166 frames). Plotted as a frequency distribution and a scatter plot (inset). D. Percentage of missing data in wild-type *Drosophila* after tracking (*n* = 29 videos, 15,166 frames). E. Frequency distribution of the deviation (in pixels) between computationally and manually derived leg tip positions (*n* = 106 frames, 636 leg tips from two videos). F. Segmentation F_0.5_ (*P* < 0.01), precision (*P* < 0.05), and recall (*P* < 0.01) scores improved for each video after learning and application of a FLLIT leg classifier, compared to using morphological parameters alone. (*n* = 8 videos, 2–3 images per video). *P* values were calculated using a nonparametric Wilcoxon matched-pairs signed rank test (learned versus morphological for each data set). Bars represent the means and standard deviations. See also S1 Video. Underlying data can be found in S1 Data. FLLIT, Feature Learning-based Limb segmentation and Tracking.

In our segmentation task, we value both precision (% of pixels FLLIT identifies as leg that are indeed leg pixels) and recall (% of actual total leg pixels in each frame, that FLLIT is able to identify as leg). The F score is a measure of both precision and recall. We opted to use F_0.5_ as it assigns a higher weight to precision than recall, thus diminishing the influence of false positive pixels, which can cause misidentification errors during tracking (see next section). We were willing to accept the tradeoff of more false negatives and missing data. To determine an optimal classification confidence threshold, we manually identified leg pixels in video frames of wild-type flies and examined classifier performance at different thresholds (S2A Fig). A threshold of 0.65 was selected for subsequent tracking analyses, based on the F_0.5_ scores for the tested classifiers, which peaked at 0.6–0.65. We then interrogated the level of similarity that can be expected between leg-tip annotations of the same set of images made by two individuals, since humans can do this task well. Pixel deviation was measured as the Euclidean distance between the pixels selected by the two individuals (Fig 2A). We found that different individuals asked to identify leg claw positions in the same set of images located the same pixels (0 pixel deviation) about 33% of the time (Fig 2B). The majority of tips were located 1–2 pixels apart. At our recording resolution, this deviation corresponded to approximately 0.75%–1.5% of body length, which averaged 133 pixels (S2B Fig).

We then compared the leg claw positions reported by the algorithm to those determined by manual annotation. We found three categories of errors: 1) misidentification errors, 2) missing data of occluded or visible legs, and 3) deviation errors. These errors are detailed below.

#### Misidentification errors

Misidentification errors occurred when leg identities were erroneously assigned to the wrong body part during tracking (after learning and leg segmentation), which can lead to tracking errors in subsequent frames. For example, in some strides, the forelegs may become occluded during leg retraction. Occluded objects cannot be detected using silhouette imaging. When trying to track the occluded leg claw, the algorithm may falsely label the antenna or another leg (S2C Fig). These errors are salient, and their correction can prevent perpetuation of the error through the rest of the video. We therefore corrected these errors before examining the rest of the tracked data (S2C Fig). Absent leg claw positions that resulted from corrections were considered as missing data (next section). By this approach, we found that wild-type flies required an average of approximately 1.6 corrections for misidentifications per 1,000 frames. 72% of videos required no corrections for misidentifications (Fig 2C).

We used FLLIT-automated background subtraction for all data sets examined for ground truth and noticed that inefficient background subtraction—which can cause poor segmentation—contributed to misidentification errors. This can arise if the fly had no/little walking motion for a significant portion of the video sequence. Poor background subtraction is typified by visible grey traces of the fly silhouette (S2D Fig). In these cases, loading of a background image generated by splicing together the first and last frames of the video, removing the fly, can improve segmentation and tracking (S2D Fig).

#### Missing values

Missing leg claw data occurred as a result of occlusion or failure to track visible legs. An average of approximately 3.6% of leg claws per video were not tracked (Fig 2D). The majority (approximately 86%) of missing values occurred in the fore legs, which were partially (28%; 235/833 missing tips; e.g., S2E Fig) or completely (72%; 598/833 missing tips, e.g., S2Ci Fig) obscured by the body when they retracted during each stride. Occasionally, hind legs were obscured by the wings. Since leg tip positions are learned from training examples, when the fore legs retract during walking, these images contain low confidence training examples in the fore legs (Fig 1Bi), which are therefore not used for learning. The fewer high-confidence training examples for the fore legs likely led to a larger percentage of missing data for this leg pair. Overall, approximately 8.1% leg-tip data for the front legs were unreported, compared to 0.01% and 2.9% for the mid and hind legs, respectively (Fig 2D).

#### Deviation errors

Deviation errors occurred when the predicted leg-tip positions differed from the ground truth annotated positions and were determined after correction of misidentification errors. Almost 98% of reported tip locations were accurate to within 3 pixels (Fig 2E). Of note, deviations of up to 3 pixels occur during manual annotation (Fig 2B), and leg tips spanned approximately 3 pixels in width under our recording parameters (Fig 2A). Therefore, in determining the accuracy of tracking, we paid special attention to errors that deviated more than 3 pixels from the manually annotated positions.

#### Effect of learning on segmentation and tracking accuracy

We quantified the effect of learning on leg segmentation and tip tracking performance, compared to solely using morphological operations (as was used to derive the training set, Fig 1Bi). As we selected only pixels of high confidence/precision for the initial training set (Fig 1Bi), before learning, recall of leg pixels was low, while precision was high (Fig 2F). After learning, recall scores showed stark improvement (*P* < 0.01), demonstrating that the algorithm was able to generalize and classify additional leg pixels based on the original training set (Fig 2F and S2 Fig). This increase in recall was accompanied by an improvement in precision (*P* < 0.05; Fig 2F). We then examined tracking performance after learning. The percentage of missing data decreased by approximately 31.5% on average for each sample (*P* < 0.0001; S2G Fig). Therefore, leg tips that would be missing if tracked using a purely morphological approach (as shown in Fig 1Bi), could sometimes be found after learning from a training set that included other training images (S2G Fig). Of the found tips, deviation errors also decreased slightly (S2H Fig).

In summary, our machine learning method can accurately track leg-claw positions of freely walking, unmarked wild-type flies from high-speed video, and is more accurate than using morphological parameters alone.

### FLLIT robustness assessment

Image analysis software should be tolerant of deviations in recording parameters, as it is challenging to identically replicate a video recording setup from one lab to another. We examined the effect of altering the following parameters on FLLIT performance: 1) image contrast: low, default, and high infrared light intensity; 2) resolution: 9, 10, and 12 mm field of view; and 3) video capture speed: 250, 500, and 1,000 frames per second (fps) (S3A Fig). Upon altering video contrast, resolution and capture speed, the percentage of missing values ranged from 2.1%–5.6% (S3Bi Fig). The number of corrections needed to re-label misidentified legs ranged from approximately 0–9.3 per 1,000 frames (S3Bii Fig). We then analyzed the final reported leg claw positions. In all conditions, approximately 97.5% of the computationally identified claws were within 3 pixels of the manually annotated positions (S3Biii Fig). These data show that FLLIT can automatically generate classifiers to track and analyze videos recorded under a range of settings and is not dependent on a stringent set of conditions or annotated training set.

As FLLIT is not rule based, we asked whether it could also be used to automatically track leg movements in other arthropods. As a test, we chose the *Myrmaplata plataleoides* salticid spider, which has eight legs and measures approximately 13 mm in length and thus differs markedly from *Drosophila* in body plan and proportions (S3C Fig). Salticid leg misidentifications occurred when legs touched or crossed over (mean = 1.2 corrections/1,000 frames; *n* = 9 videos, 12,683 frames, 101,464 legs) (S3B Fig). These corrections resulted in an average of approximately 0.66% of missing leg data (S3C Fig). Computationally predicted leg-tip positions compared favorably with manual annotation; >99% of the tracked data deviated by <3 pixels from user-annotated positions (S3D Fig). A small fraction deviated by >6 pixels, which was due to legs touching or crossing over during walking. In summary, these data suggest that FLLIT can be used to accurately track leg tips in other arthropods (S2 Video).

### Side-by-side performance comparison

**Fig 3:**
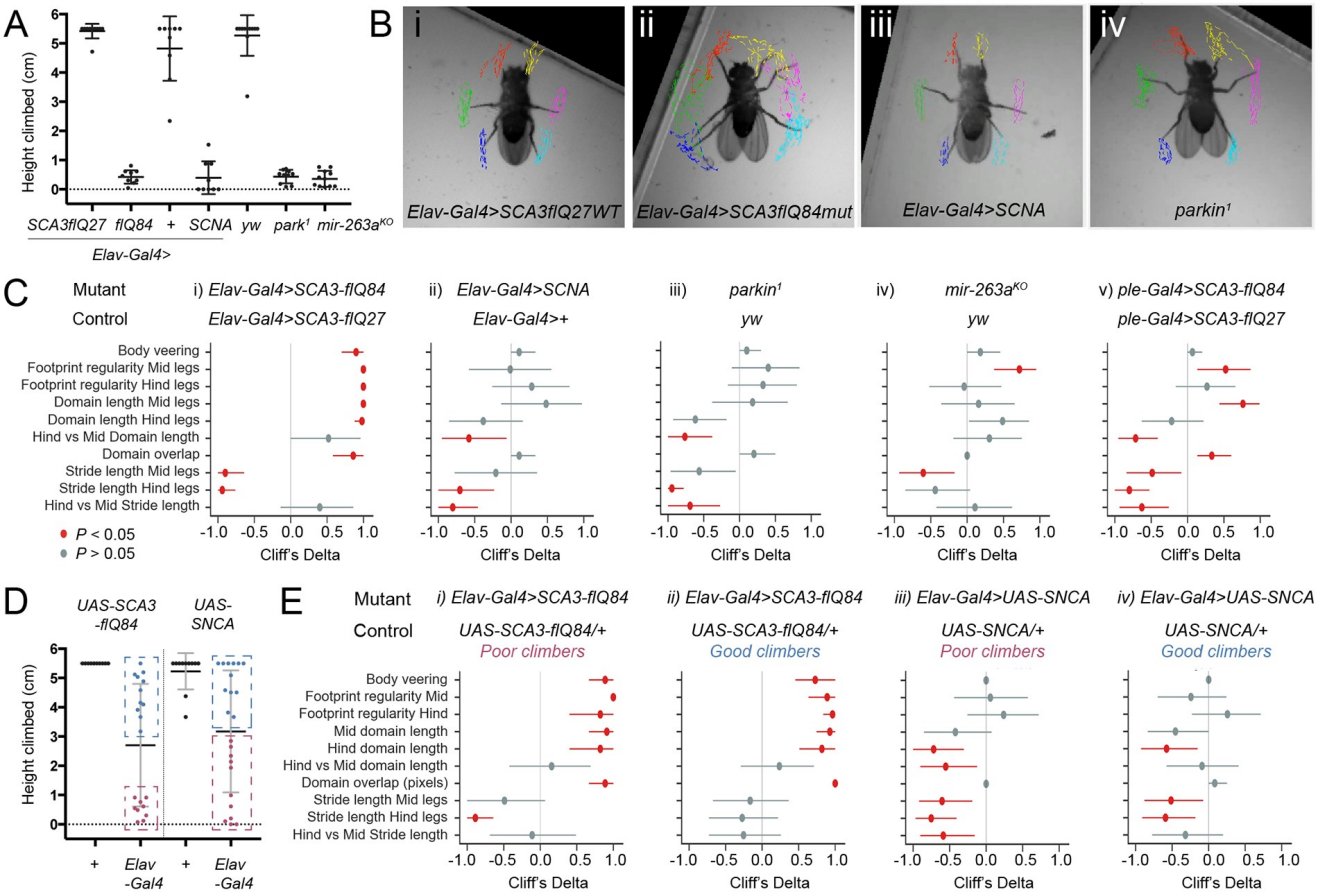
Gait signatures of *Drosophila* models of neurodegeneration reveal properties of underlying neuronal dysfunctions. A. Climbing performance (highest height climbed in 30 s) of flies analyzed. Representative FLLIT-derived walking leg traces of the respective genotypes. C. Cliff’s delta indices of effect sizes (filled circles) of SCA3 and PD-relevant gait parameters from Table 2, with 95% confidence intervals (horizontal lines), with respective *P* values. Positive Cliff’s delta for a given parameter indicates an increase in mutant flies compared to respective controls, whilst negative Cliff’s delta indicates a decrease. Detailed statistics are given in S1 Table. Raw values are plotted in S5 Fig. The following gait parameters were analyzed: body veering (number of body turns normalized to the average number of strides per leg), footprint regularity (standard deviations of the anterior extreme position, normalized to body length), leg domain lengths (normalized to body length), Average ratio of the hind vs mid domain length of the right and left sides, Domain overlap (number of pixels overlapping between leg domains, normalized to the average number of strides per leg), Stride lengths of the mid and hind legs (normalized to body length), Average ratio of the hind vs mid stride lengths of the right and left sides. Body length measurements were individually obtained for each fly for normalization. Genotypes examined: *Elav-Gal4>UAS-SCA3-flQ27* (*n* = 10), *Elav-Gal4>UAS-SCA3-flQ84* (*n* = 10), *Elav-Gal4>+* (*n* = 9), *Elav-Gal4>UAS-SNCA* (*n* = 9), *yw* (*n* = 11), *park*^*1*^ (*n* = 10), *mir-263a*^*KO*^ (*n* = 11), *ple-Gal4> UAS-SCA3-flQ27* (*n* = 14), *ple-Gal4> UAS-SCA3-flQ84* (*n* = 15). D. Climbing performance (highest height climbed in 30 s) of *Elav-Gal4>UAS-SCA3-flQ84*, *Elav-Gal4>UAS-SNCA*, and respective *UAS/+* control flies. Hashed boxes demarcate the approximately 10 poorest (red boxes) and approximately 10 best (blue boxes) climbers showed in panel E. Genotypes examined: *UAS*-*SCA3-flQ84/+* (*n* = 10), *Elav-Gal4>UAS-SCA3-flQ84* (*n* = 9 poor climbers, and *n* = 11 good climbers, the best climbers in the population), *UAS-SNCA/+* (*n* = 11), *Elav-Gal4>SCA3-flQ84* (*n* = 11 poor climbers and *n* = 12 good climbers). E. Cliff’s delta indices of flies from panel D, comparing the poor and good climbers for *Elav-Gal4>UAS-SCA3-flQ84* and *Elav-Gal4>UAS-SNCA* to their respective UAS/+ controls. *P* values were calculated using a non-parametric Mann-Whitney test except for *park*^*1*^ and *mir-263a*^*KO*^, which shared the same control (*yw*); hence, *P* was calculated using a nonparametric Kruskal–Wallis test with Dunn’s multiple comparisons posthoc test (see S5 Fig). See also Table 2 and S3–S7 Videos. Underlying data can be found in S1 and S2 Data. FLLIT, Feature Learning-based Limb segmentation and Tracking; PD, Parkinsons Disease; SCA3, Spinocerebellar ataxia Type 3; SNCA, alpha-synuclein.

Two types of approaches are currently state-of-the-art for *Drosophila* leg movement tracking. One uses thresholding and dynamic masking methods (TDM) to automatically identify leg tips [22,24], while two recent studies employ deep learning algorithms [25,26] using user-annotated training sets. We first compared the performance of FLLIT against TDM-based software provided by Isakov and colleagues [22]. We found that the TDM method was sensitive to image contrast and required flies to walk sufficiently close to the center of the video for thresholding. Hence, videos had to be cropped by trial and error for successful tracking. We directly compared the tracked positions, without making any error corrections for either method. The percentage of missing values when using either method was comparable (S4A Fig). However, 16.1% of positions identified using TDM were located >3 pixels from the manually-derived positions, compared to 1.2% for FLLIT (>13 fold difference) (S4B Fig). (A 3 pixel threshold was chosen as manually-annotated claw positions do not deviate more than 3 pixels from one another, and leg claws spanned approximately 3 pixels in width under our recording parameters.) (Fig 2A and 2B) FLLIT also performed markedly better than TDM under other recording settings (S4C Fig). Of note, we could not find parameters that allowed low contrast images to be tracked using TDM; these were accurately tracked by FLLIT (Fig 3, S4A and S4C Fig). To gauge the usefulness of a tracking tool, the magnitude of the deviation errors may matter less than the rate of error correction required. Hence, we assessed what percentage of frames required user correction (deviations >3 pixels; see text for Fig 2). By the TDM method, approximately 32%–63% of frames contained at least 1 one leg that deviated >3 pixels from the manually-derived positions, compared to 2.8%–8.1% when using FLLIT (a 4–22-fold difference) (S4D Fig).

A deep-learning method for movement tracking, DeepLabCut, was recently published [25]. While FLLIT and the TDM method require no user input, DeepLabCut requires the user to pre-train the algorithm using at least 200 to thousands of user-annotated images. We compared the performance of DeepLabCut to FLLIT in our leg tracking task. When DeepLabCut was trained on a random subset of images (200 frames) from a video of about 500 frames, and used to test on novel images from the same video, 28.1% of frames predicted using DeepLabCut contained at least one leg deviating >3 pixels, compared to 6.3% with FLLIT (S4D Fig). When DeepLab Cut was trained on images from one video, and then used to track a different video taken under similar settings, 100% of frames contained at least one leg deviating >3 pixels, even when we manually matched predicted leg claw positions to the closest leg (i.e., using DeepLabCut to find leg claw positions, without requiring labelling of leg identity). We therefore conclude that FLLIT produces more accurate data on this task than these state-of-the-art methods, while not requiring any user annotation of training sets.

### Characterization of gait in *Drosophila* models of SCA3 and PD

Ataxic gait in SCA3 is typified by body veering, erratic foot placement, leg crossing over, lurching steps, and intention/action tremor [5,31], while gait in PD patients is marked by rigid, shuffling steps, and resting tremor [1,31] (Table 2). Therefore, these two diseases exhibit distinctly different gaits that arise from their underlying etiologies. We used FLLIT to determine these corresponding gait characteristics in previously described *Drosophila* models of the two diseases (Table 2).

**Table 2:** Gait features of PD and SCA3 and corresponding gait parameters computed by FLLIT.

	Gait features of disease				
SCA3	Veering	Erratic foot placement and leg crossing over	Lurching steps	Short strides	Action tremor
PD	Not a feature	Not a feature	Leg rigidity	Rigidity, Shuffling	Resting tremor
Measurement Parameter	Number of body turn events	Footprint regularity	Size of leg domains, degree of domain overlap	Stride length	Irregularities in leg traces

**Abbreviations**: FLLIT, Feature Learning-based Limb segmentation and Tracking; PD, Parkinsons Disease; SCA3, Spinocerebellar ataxia Type 3

For SCA3, body veering would be reflected by an increased number of body turn events, while erratic foot placement and leg crossing over would result in large deviations in footprint regularity [19]. Lurching steps would be reflected by enlarged leg domains (traced out trajectories of each leg path; Table 1) with greater overlap, short strides by decreased stride length, and action tremors by irregularities in the leg traces. For PD, leg rigidity would be associated with shorter strides and possibly smaller leg domain sizes, assuming a low deviation in footprint regularity (Table 2).

To model SCA3, we used the pan-neuronal driver *Elav-Gal4* to express in all neurons either wild-type SCA3/Ataxin-3 with a normal number of polyglutamine repeats (SCA3 full-length [fl] Q27) or mutant SCA3 with an expanded number of repeats (SCA3 fl Q84) [32,33]. For PD we examined two models: Expression of wild-type human alpha-synuclein (SNCA) under control of *Elav-Gal4* [14, 34] (elevated levels of wild-type SNCA in both flies and humans cause neurodegeneration [14,35]), and homozygous *parkin* mutant flies [36–40] (mutations in *parkin* underlie the most common cause of early onset PD [41]). These mutants exhibit neurodegeneration and gross motor defects reflected in poor climbing ability [14,32–34,36–40]. To enable comparison of gait defects amongst all the different genotypes, we used climbing ability as a readout of phenotypic severity, at first selecting for analysis mutant flies that exhibited similar climbing performance (Fig 3A). Flies that climbed between 0.3–0.9 cm were used for recording and gait analysis, which accounted for approximately 20%–30% of the population depending on the genotype (additional details in Methods). For two mutants, *Elav-Gal4>SNCA* and *Elav-Gal4>SCA3-flQ84*, we also compared the gait signatures of good climbers against poor climbers (see below).

**Fig 4:**
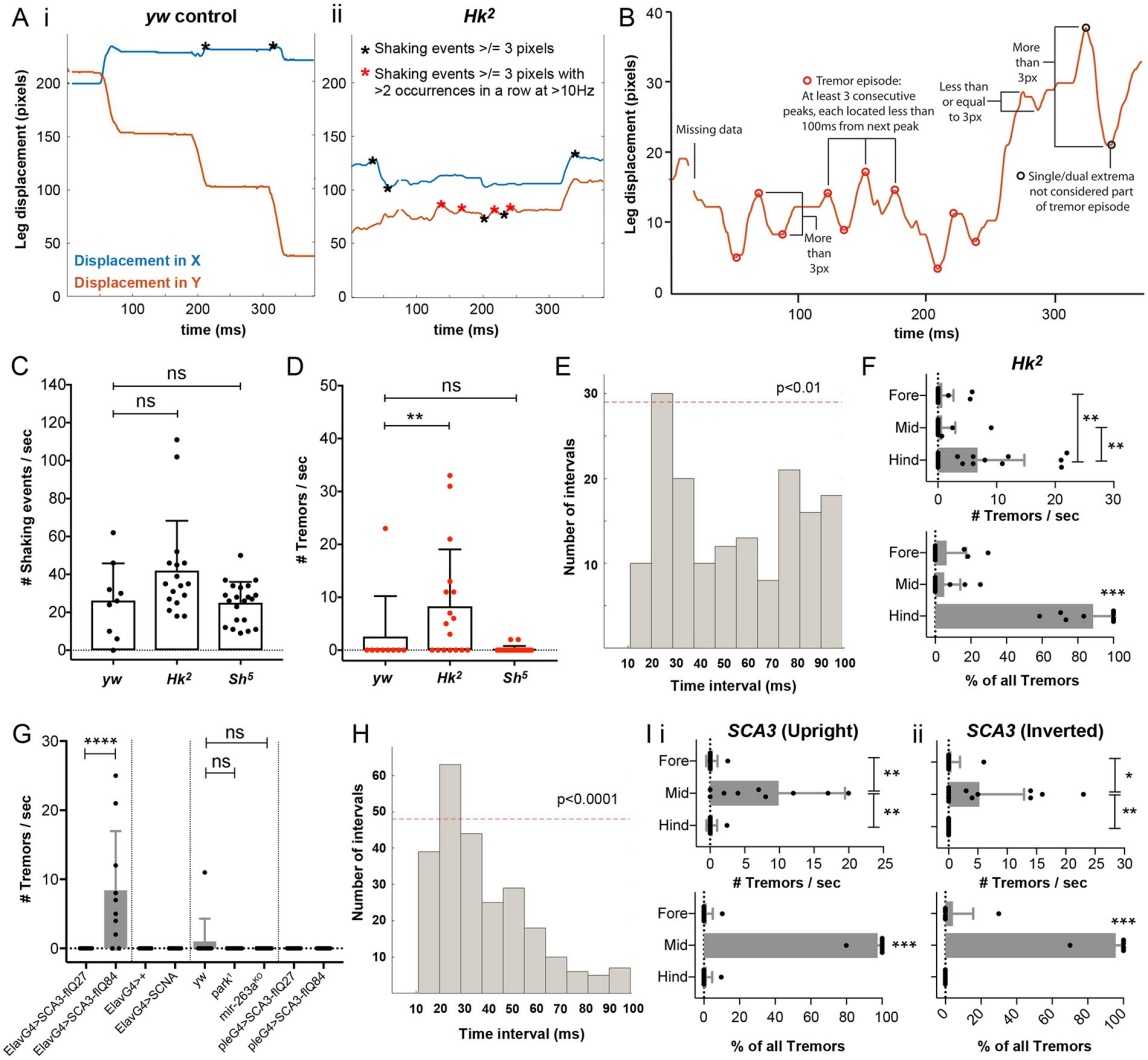
Detection and characterization of high-frequency leg tremors in *Drosophila* mutants. A. Representative leg traces of freely walking control (*yw*) and *Hk*^*2*^ mutant *Drosophila*. B. Schematic showing the parameters used to determine tremor events. C, D. Number of shaking (C) and tremor (D) events in control (*n* = 11), *Hk*^*2*^ (*n* = 17), and *Sh*^*5*^ (*n* = 21) *Drosophila*. E. Distribution of the time interval durations between tremor peaks or valleys in *Hk*^*2*^ flies. A significant proportion of events showed an interval duration of 20–30 ms (*P* < 0.01; *P* value was determined by running a nonparametric permutation test with 100,000 iterations), reflecting a tremor frequency of approximately 33–50Hz. F. Top: number of tremors/s in the fore, mid, and hind legs of each *Hk*^*2*^ fly (*n* = 17). Bottom: percentage of all tremors accounted for by either the fore, mid or hind legs in each *Hk*^*2*^ fly that exhibited tremors. G. Number of tremors per second exhibited by each of the genotypes examined: *Elav-Gal4>SCA3-flQ27* (*n* = 10), *Elav-Gal4>SCA3-flQ84* (*n* = 10), *Elav-Gal4>+* (*n* = 9), *Elav-Gal4>SCNA* (*n* = 9), *yw* (*n* = 11), *park*^*1*^ (*n* = 10), *mir-263a*^*KO*^ (*n* = 11), *ple-Gal4>SCA3-flQ27* (*n* = 14), and *ple-Gal4>SCA3-flQ84* (*n* = 15). H. Distribution of the time interval durations between tremor peaks or valleys in *Elav-Gal4>SCA3-flQ84* flies. A significant proportion of events showed an interval duration of 20–30 ms (*P* < 0.0001), reflecting a tremor frequency of approximately 33–50Hz. I. Top: number of tremors/s in the fore, mid, and hind legs of each *Elav-Gal4>SCA3-flQ84* fly when (i) walking upright (*n* = 10) or (ii) walking inverted (*n* = 15). Bottom: percentage of all tremors accounted for by either the fore, mid, or hind legs in each *Elav-Gal4>SCA3-flQ84* fly that exhibited tremors when (i) walking upright (*n* = 8) or (ii) walking inverted (*n* = 7). *P < 0.05, **P < 0.01, ***P < 0.001, ****P < 0.0001. All data were analyzed using a nonparametric Kruskal–Wallis test with Dunn’s multiple comparisons posthoc test unless otherwise stated above. Bars represent the means and standard deviations. See also S8 and S9 Videos. Underlying data can be found in S1 Data. SCA3, Spinocerebellar ataxia Type 3; SNCA, alpha-synuclein.

Leg tracking with FLLIT showed that flies expressing wildtype human SCA3-flQ27 in all neurons walk coordinately, with strides that form regular leg domains (Fig 3Bi and S3 Video). However, pan-neuronal expression of SCA3-flQ84 mutants led to a strikingly aberrant gait (Fig 3Bii and S4 Video). Similar to that seen in SCA3 patients [5,31], SCA3 flies exhibited repeated veering and lurching, detected as body turns (Fig 3Ci and S5Ai Fig). These uncoordinated movements and erratic foot placement resulted in poor footprint regularity [19], reflected in large standard deviations of the anterior extreme positions (AEP; Fig 3Ci and S5Aii Fig) of the mid and hind legs and longer (Fig 3Ci and S5Aiii Fig) leg domains of both the mid and hind legs. These leg domains were so large as to intersect with one another (Fig 3Bii and 3Ci, and S5Aiv Fig). A similar gait was observed for mutant SCA3 flies when compared to UAS-SCA3-flQ84/+ controls (Fig 4Ei and 4Eii).

While these phenotypes were striking in their similarity to cerebellar ataxic gait [42], they could result from nonspecific toxicity due to pan-neuronal expression of a pathogenic protein that is not endogenously expressed in *Drosophila* (SCA3/Ataxin-3-flQ84). We therefore examined gait in flies expressing SNCA, the hallmark protein that accumulates in Lewy bodies in PD, which is also not endogenously expressed in *Drosophila*. *Elav-Gal4*-mediated expression of wild-type SNCA was previously shown to cause neurodegeneration and climbing defects [14]. We used a recently published codon-optimised version of UAS-SNCA with high Gal4-driven expression [34]. If both SNCA and SCA3 expression cause non-specific toxicity effects, flies of each genotype with similar climbing performance (Fig 3A) should exhibit similar gait profiles. However, SNCA-expressing flies did not exhibit hyperkinetic movements of increased body turning, low footprint regularity, enlarged leg movement domains nor aberrant domain overlap (Fig 3Biii and 3Cii, S5Bi–S5Bv Fig, and S5 Video). To the contrary, they showed some traits of hypokinetic movements, especially in the hind legs: leg rigidity in the form of short strides and leg domain lengths that trended slightly shorter in the hind legs, relative to the mid legs (Fig 3Biii and 3Cii, S5Biii–S5Bvii Fig, and S5 Video). A similar gait was observed for mutant SNCA flies when compared to UAS-SNCA/+ controls (Fig 4Eiii and 4Eiv). Therefore, interestingly, expression of two different non-endogenous human disease proteins in all fly neurons not only result in markedly distinct gaits, but these gaits reflect characteristics observed in the respective human diseases.

Dopaminergic neurons preferentially degenerate when SNCA is expressed in fly neurons, and their loss is implicated in the associated climbing defects [14]. We therefore asked whether other PD models that exhibit dopaminergic neuron degeneration show a similar gait profile. We examined a mutant of *parkin*, a conserved ubiquitin ligase that is one of the most common mutations underlying familial PD [38,41,43] (S6 Video). Interestingly, *park*^*1*^ mutant flies also showed a decrease in hind leg domain length (Fig 3Biv and 3Ciii, and S5Ciii Fig) and hind leg stride length compared to the mid legs (Fig 3Biv and 3Ciii, and S5Cvi Fig). This resulted in lowered ratios of hind to mid leg domain length (Fig 3Ciii and S5Civ Fig) and stride length (Fig 3Ciii and S5Cvii Fig). Overall, the PD models *park*^*1*^ and *Elav-Gal4>SNCA* showed gait profiles that were strikingly similar (Fig 3Cii and 3Ciii), despite being genetically dissimilar. We wondered if relative hind leg rigidity was a basal property of poor motor function/climbing ability and hence examined *mir-263a*^*KO*^ flies, which also climb poorly [18] (Fig 3A). *Mir-263a*^*KO*^ flies did not exhibit preferential hind leg rigidity (Fig 3Civ and S5C Fig), and its gait signature differed from that of SCA3 and PD flies (Fig 3Ci–3Civ).

That SNCA and *park*^*1*^ flies shared a common gait signature led us to hypothesize that dopaminergic neuron dysfunction may be a common underlying cause. To test this, we expressed mutant SCA3 under control of the dopaminergic driver *ple-Gal4*. Interestingly, while pan-neuronal expression of mutant SCA3 caused ataxic gait and hyperkinetic movements of both the mid and hind legs (Fig 3Bi and 3Ci, S5A Fig, and S7 Video), expression of mutant SCA3 with *ple-Gal4* caused short strides preferentially in the hind legs. Mid leg domains were enlarged but not the hind leg domains, leading to a decrease in the hind/mid leg domain length ratio (Fig 3Cv and S5D Fig). The lack of decrease in the hind leg domain length despite the much shorter hind leg strides could be due to the larger deviations in footprint regularity, which would tend to enlarge leg domains. Overall, expression of mutant SCA3 in dopaminergic neurons led to a gait signature that more closely resembled that of *park*^*1*^ and *Elav-Gal4>SNCA* flies (Fig 3Cv versus Fig 3Cii and 3Ciii). These data suggest that perturbation of dopaminergic neuron function in *Drosophila* is associated with rigid, short hind leg strides.

Mutant flies of the same genotype can show a wide range of climbing performance (Fig 3D). We had chosen similarly poor climbers of each genotype to enable comparison between the different genotypes, as we had anticipated that gait defects may also show variability. We now wondered whether good climbers would also exhibit aberrant gait and hence examined gait signatures of poor vs. good climbers (the approximately 10 best and worst climbers in the population) for SCA3 and SNCA flies (Fig 3D; tested flies cover approximately 80% [*ElavGal4>UAS-SCA3-flQ84*] and 100% [*ElavGal4>UAS-SNCA* and both *UAS-only* controls] of the population of flies randomly sampled in a climbing assay). Interestingly, we found that the gait profiles for poor and good climbers were similar (Fig 4Ei versus 4Eii and Fig 4Eiii versus 4Eiv), although the SNCA poor climbers had a slightly stronger phenotype than the good climbers. This suggests that gait analysis can reveal defects even in flies that climb well.

### Detection and characterization of high frequency leg tremors in freely moving *Drosophila*

Tremor is defined as an involuntary, rhythmic, oscillatory movement of a body part [44]. To determine if FLLIT could detect and quantify leg tremors in freely walking flies, we first examined flies known to tremble: *Shaker* (*Sh*^*5*^) and *Hyperkinetic* (*Hk*^*2*^) mutants, which carry lesions in alpha and beta subunits of the Shaker voltage-gated potassium (Kv) channel and exhibit leg shaking under ether anesthesia [45]. Aged, freely walking *Sh* mutants were described to exhibit “quivering” behavior [17], but the characteristics of these movements have not been previously quantified.

Compared to aged control (*yw*) flies (S8 Video), *Hk*^*2*^ mutants appeared to exhibit occasional tremor-like movements (S9 Video). As such, wild-type legs followed relatively smooth paths, whereas leg paths of *Hk*^*2*^ mutants were relatively irregular and uneven (Fig 4A). The number of rhythmic repeats of movements per second gives the tremor frequency. In humans, tremor frequency is usually categorized as <4, 4 to 8, 8 to 12, and >12 Hz [44]. Hence, we quantified the number of shaking movements, as well as the frequency of these movements by charting the time interval between shaking movements.

To first quantify the number of shaking movements, local extrema of at least 3 pixels in amplitude were identified from the traces (circles and stars; Fig 4A and 4B). A cutoff of 3 pixels was chosen to filter out small displacements that occur due to tracking errors (since approximately 98% of tracked positions were within 3 pixels of the actual leg claw as located by manual annotation; Fig 2E) and roughly corresponded to the width of a leg tip (Fig 2A). *Hk*^*2*^ mutants exhibited approximately 42 shaking events/s on average, whilst wild-type controls and *Sh*^*5*^ mutants exhibited approximately 25 shaking events. However, this was not statistically significant (Fig 4C). As tremors are periodic shaking movements, we then looked for tremor-like episodes during walking: a series of three or more shaking events separated by <100 ms (conservatively chosen based on the average stride rate of approximately 10 Hz in control flies in our assay; Fig 4Ai). Each of these events was defined as a tremor event (red circles and stars; Fig 4A and 4B). *Hk*^*2*^ flies exhibited an average of approximately 8 tremor events per second, significantly more than control and *Sh*^*5*^ flies, which showed almost no tremor (*P* < 0.01) (Fig 4D).

We estimated the frequency of *Hk*^*2*^ leg tremors by examining the time interval between consecutive tremor events. A predominant inter-peak interval of 20–30 ms was observed (*P* < 0.01 by running a permutation test with 100,000 iterations; Fig 4E), corresponding to a tremor frequency of approximately 33–50 Hz. Interestingly, *Hk*^*2*^ leg tremors mostly occurred in the hind legs: An average of 6.7 tremor events per second were observed in hind legs of *Hk*^*2*^ flies, accounting for approximately 89% of the tremors in each fly on average (Fig 4F). Therefore, our method is able to detect and measure rapid, tremor-like movements in *Drosophila*.

We then quantified tremor behavior in the neurodegeneration mutants that we previously examined for gait defects (Fig 3). We found that *SCA3* mutants were the only flies to show tremor amongst the genotypes examined (Fig 4G and S4 Video). PD flies did not show tremors when walking nor did flies expressing SCA3 only in dopaminergic neurons (Fig 4G). *Elav-Gal4>SCA3-flQ84* tremor showed a similar frequency to that of *Hk*^*2*^ flies (*P* <0.0001 by running a permutation test with 100,000 iterations; Fig 4H). However, unlike *Hk*^*2*^ flies, 95% of *SCA3* tremors occurred in the mid legs: An average of 9.9 tremor events per second were observed in mid legs of *Elav-Gal4>SCA3-flQ84*flies, accounting for approximately 97.5% of the tremors in each fly on average (Fig 4Ii). A similar trend held when *Elav-Gal4>SCA3-flQ84* mutants flies walked upside-down/inverted—an average of 5.3 tremor events per second were observed in mid legs of inverted *Elav-Gal4>SCA3-flQ84* flies, accounting for approximately 95.7% of the tremors in each fly on average (Fig 4Iii). This suggests that there are at least two different motor circuits whose perturbation can cause tremor.

## Discussion

Our study describes the development of FLLIT, a fully automated machine-learning method for tracking leg claw positions of freely moving flies and its use to study gait and tremors in fly models of PD and SCA3. FLLIT has two distinct features: first, it exhibits a high level of accuracy that allows it to measure movements as fine as tremors, and second, it does not require any user-annotation of training sets. Our results show that machine learning can improve upon an approach that uses only morphological parameters, so that supervised learning is carried out on machine-derived training sets, i.e., the algorithm trains itself without human input. As training sets are derived from the test video itself, training samples are very similar to test samples, possibly explaining the high level of accuracy that FLLIT achieves. This approach can be used to customize solutions for which training data is scarce or for which there is great variability in recording parameters, such that to collect a representative training set would require an unfeasibly large number of manually annotated training samples. The FLLIT method is fully automated and requires no manual curation of data at any point, other than the checking and correction, if any, of the final tracked data. For this final step, FLLIT is more accurate than the other methods that we made comparisons to (S4 Fig). The number of corrections required will depend on the types of gait defects in the genotypes of interest. Legs that touch or cross over during walking may require a larger number of corrections.

FLLIT does not require a contact surface for detection or leg markers for tracking, is not sensitive to variations in recording parameters, and is not rule based. These strengths permit its application to other animals, which we show by application to spiders. Images of segmented legs are saved by FLLIT and could potentially be used for subsequent analyses. Tracked FLLIT data may be applied to other methods that use tracked and/or labeled data to identify and describe complex behaviors and patterns [46–48] or combined with methods for circuit manipulation and functional imaging. While FLLIT achieves great accuracy in our walking task, it is customized for studying gait and tremors and is therefore not optimal for all applications. The semiautomatic FlyLimbTracker [23], while more time-consuming than necessary for tracking only limb tips, could be more suitable if tracking of other leg segments is required. The FlyLimbTracker strategy could also be combined with leg segmentation images produced by FLLIT for improved automation of leg segment tracking. FlyWalker [19] may be more suitable than FLLIT if only footprint information was required. Recently developed deep learning approaches [25, 26] allow for versatile tracking of varied behaviors and setups based on user-annotated training sets. Combining both deep learning approaches and FLLIT’s strategy of “self-training” by adapting partially available morphological markers may lead to further optimization and improved automation of machine vision algorithms.

Using FLLIT, we characterized, for the first time, specific gait and tremor dysfunctions in fly neurodegeneration mutants. These mutants would be undifferentiable using classic climbing or locomotor assays, although it would be interesting to test them in a recently reported sensitive climbing assay [49]. Gait characterization allows comparison to human movement disorders, for which gait parameters measured in the clinic can be used to aid disease diagnosis. Due to the pixel resolution of our camera, for accurate tracking, we were limited to a field of view of about 10 x 10 mm. We note that this small arena could restrict the fly’s movements, and bias data for turning. In future, we could allow the fly to walk in a larger arena while restricting the field of view of the camera. With this caveat in mind, we were surprised to find that gait in SCA3 and PD flies shared common characteristics with that observed in the respective human diseases. SCA3 flies exhibit lurching, ataxic gait, while PD flies show stride rigidity, preferentially in the hind legs. We do not know why the hind legs were especially affected. These phenotypes are not differing in degree, rather, they are opposite in character: The former being hyperkinetic, and the latter, hypokinetic. Our study also suggests that, just like in humans, perturbation of dopaminergic circuits underlie the PD fly gait dysfunctions, resulting in a similar gait signature in PD fly models that were otherwise genetically unrelated. By controlling the neurons in which a mutant transgene was expressed, we were able to induce a PD-like phenotype using a mutant SCA3 protein. This demonstrates that the movement phenotype depends on which neurons were affected, rather than the specific nature of the mutation. In future studies, it could be interesting to compare whether and how the effect of SNCA in dopaminergic neurons differs from that of SCA3-flQ84 in these same neurons. The molecular events that cause specific neurons to be affected when the mutant transgenes were expressed in all neurons will also be an interesting avenue for future study. The conservation of movement phenotypes caused by dopaminergic dysfunction suggests there may be common fundamental principles of motor control and circuitry between flies and mammals that manifest during disease. The powerful tools and approaches available in the fly can now be used to try to dissect the underlying mechanisms. Going forward, manipulation of other subsets of neurons may help us to understand how specific behavioral dysfunctions can result from perturbation of disease genes in different circuits. Classification of mutants with similar gait signatures may reveal novel relatedness of disease pathways and molecular mechanisms.

Tremor is increasingly prevalent in our aging population [50], yet, we know very little regarding the mechanisms that underlie such movements as there is no good animal model for tremor. In this study, we detected and quantified leg tremor in freely moving *Drosophila*, from large data sets of leg movement data tracked from high-speed video. To our knowledge, this is the first automated, computer vision-based method for tremor measurement in any animal model. We propose that by combining these data with functional and imaging-based genetic tools, *Drosophila* models will be useful for understanding how tremor-like movements can occur. Our analysis of *Hk*^*2*^ and SCA3 mutants indicated that fly tremors occurred at 30–50 Hz. This is much more rapid than the tremors that occur in humans, such as those in Essential Tremor (4–12 Hz [51]), and orthostatic tremors (13–18 Hz [52]). The reason for this can only be determined when the biomechanical and cellular mechanisms underlying tremor are better understood. Our study only measured tremors that occurred during walking, which are likely action tremors. Several other types of tremor exist in humans, including resting and postural tremor [53]. Notably, the PD flies examined did not exhibit action tremors. We also did not observe resting tremors in these flies; however, different assay conditions may need to be developed to systematically determine if other categories of tremor found in humans also occur in *Drosophila*. It will be now interesting to examine other fly mutants that exhibit tremor, to determine their different tremor “signatures” and to compare these to the corresponding human diseases.

## Methods

### Iterative training module for leg segmentation

Leg segmentation is achieved using a supervised learning approach. Training images are automatically generated via image-processing steps, without user annotation.

First, a pool of representative images is obtained from a set of input images. In cases where the image set is an entire video, the representative images are obtained by uniform sampling from the video frames. To this aim, we select one image frame from every 20 frames. The operations described below are carried out for each image in the pool.

1. The silhouette of the subject animal (*Drosophila*) is extracted as a binary foreground via background subtraction, using the following formula:
  Silhouette=|Image(x,y)-Background|>Threshold
2. Skeletonization and edge detection of the silhouette foreground (standard image morphological operations) was performed on the images. The overlap between the skeleton image and the edge image primarily occur in the leg regions. The pixels within these regions are identified as positive samples containing the leg segments. The negative image consists of the fly body and background.
3. A fixed number of high-confidence samples are extracted after morphological operations on the segmented results, and used in a supervised learning approach. Each training sample is extracted as an image patch of 41 x 41 pixels and represented as an instance label pair (***x***_i_, *y*_*i*_), where ***x***_i_ denotes the image patch and *y*_*i*_ = ±1 denotes the corresponding label of the central pixel. This process provides the initial training data set to learn the following Kernel–Boost classifier [54,55]:
  $$\varphi (x)=\sum_{j=1}^{M}\alpha_{j}h_{j}(x)$$
  in which the classifier function *φ*(*x*) is a weighted sum of *M* = 100 weak learners *h*_*j*_ with corresponding weights *α*_*j*_. The primary framework of the Kernel–Boost classifier is gradient boosting, which adopts a greedy algorithm with quadratic approximation (Box 1).

**Box 1. Greedy algorithm with quadratic approximation [56,57]**

INPUT:

  Labeled training samples {(***x***_i_, *y*_*i*_)}

  Exponential loss function $L(y_{i},\varphi {(x}_{i}))=e^{y_{i}{\varphi (x}_{i})}$

  # Iterations *M* = 100

 1. Initialize model with: *φ*_0_(·) = 0

 2. For *j* = 1: *M*

 3. Compute the weight $w_{i}^{j}={\frac{\partial^{2}L(y,\phi )}{\partial \phi^{2}}|}_{\phi =\varphi_{j-1}}$

  and the “pseudo-residual” $r_{i}^{j}=-\frac{1}{w_{i}^{j}}{\frac{\partial L(y,\phi )}{\partial \phi }|}_{\phi =\varphi_{j-1}}$

 4. Update the j-th weak learner *h*_*j*_:

$$h_{j}(\cdot )={\text{argmin}}_{h(.)}\sum_{i=1}^{N}w_{i}^{j}{\left(h(x_{i})-r_{i}^{j}\right)}^{2}$$

 5. Update the j-th weight *α*_*j*_ by line search:

$$\varphi_{j}(\cdot )=\varphi_{j-1}(\cdot )+\gamma \alpha_{j}h_{j}(\cdot )$$

 6. End for on j

OUPUT:

$$\varphi (x)=\sum_{j=1}^{M}\alpha_{j}h_{j}(x)$$

The Kernel–Boost classifier uses the following approach to update the weak learner of the algorithm (Box 1, step 4). The training set *T* is randomly split into two sets of fixed sizes:

T=T1∪T2

Instead of relying on predefined features, here features are automatically learnt on the first training set *T*1 in the form of convolution kernels. The weak learners are simultaneously learnt in the form of decision trees on the second training set *T*2. Essentially, the weak learners *h*_*j*_ will be a combination of kernels ***K*** and tree parameters *τ* (split threshold), *η*(leaf values).

### Learning kernels on T1

The kernels are square windows, 4–19 pixels in length and operate on a specific fixed square region within each image patch ***x***_*i*_. A total of 100 candidate kernels are obtained at boosting iteration *j*, with the p-th kernel ($K_{p}^{j}$) being identified by:

$$K_{p}^{j}=\underset{K}{\text{argmin}}\sum_{i\in T_{1}}{w_{i}^{j}(K{*x}_{i}-r_{i}^{j})}^{2}+\lambda_{p}\sum_{m,nneighbours}{{(K}^{\left(m\right)}-K^{\left(n\right)})}^{2}$$

Where ***K*** * ***x***_*i*_ denotes the convolution of kernel ***K*** on the fixed square region within image patch ***x***_*i*_. The second term *λ*_*p*_ Σ_*m*,*n* neighbours_(***K***^(*m*)^–***K***^(*n*)^)^2^ is a regularization term introduced to impose a smooth kernel. Here, ***K***^(*m*)^ denotes the *m*-th pixel of kernel ***K*** and *λ*_*p*_ is a regularization factor for the p-th kernel that is randomly assigned to one of three values: 100, 500, or 1,000.

### Constructing decision tree on T2

Decision tree learning is performed one split at a time, up to a depth of five levels (32 leaf nodes). To learn the tree parameters (split threshold *τ*^*j*^ and leaf values $\eta_{L}^{j},\eta_{R}^{j}$) at a decision node, a split search is first performed on T2 for every candidate kernel $K_{p}^{j}$ as follows:

$$\tau_{p}^{j},\eta_{L,p}^{j},\eta_{R,p}^{j}=\underset{\tau ,\eta_{L},\eta_{R}}{\text{argmin}}\sum_{i\in T_{2}|K_{p}^{j}*X_{i}<\tau }{w_{i}^{j}(\eta_{L}-r_{i}^{j})}^{2}+\sum_{i\in T_{2}|K_{p}^{j}*X_{i}\geq \tau }{w_{i}^{j}(\eta_{R}-r_{i}^{j})}^{2}$$

in which $\tau_{p}^{j}$ is the split threshold and $\eta_{L,p}^{j},\eta_{R,p}^{j}$ are the leaf values for a specific kernel $K_{p}^{j}$. In actual implementation, the optimal split threshold *τ*_*p*_ is found by exhaustive search. Subsequently, the leaf values $\eta_{p}^{j}$ are simply given by the weighted sum:

$$\eta_{L,p}^{j}=\frac{1}{\sum_{i\in T_{2}|K_{p}^{j}*X_{i}<\tau }w_{i}^{j}}\sum_{i\in T_{2}|K_{p}^{j}*X_{i}<\tau_{p}}w_{i}^{j}r_{i}^{j}$$

$$\eta_{R,p}^{j}=\frac{1}{\sum_{i\in T_{2}|K_{p}^{j}*X_{i}\geq \tau }w_{i}^{j}}\sum_{i\in T_{2}|K_{p}^{j}*X_{i}\geq \tau_{p}}w_{i}^{j}r_{i}^{j}$$

The split cost is evaluated for each candidate kernel $K_{p}^{j}$:

$${split\;cost}_{p}=\sum_{i\in T_{2}|K_{p}^{j}*X_{i}<\tau_{p}}{w_{i}^{j}(\eta_{1,p}-r_{i}^{j})}^{2}+\sum_{i\in T_{2}|K_{p}^{j}*X_{i}\geq \tau_{p}}{w_{i}^{j}(\eta_{2,p}-r_{i}^{j})}^{2}$$

The kernel giving the smallest split cost is chosen for this split.

Subsequently, the split search is performed recursively up to the desired tree depth and the final output for the *j*-th weak learner is the set of kernels $K_{p}^{j}$ as well as the decision tree. A summary of the parameters used is shown in Table 3.

**Table 3:** Parameters used in the Kernel–Boost algorithm.

Parameter	Value
Number of positive samples	30,000
Number of negative samples	30,000
Sample image patch size	41 square pixels
Number of samples for learning convolutional kernels	10,000
Min. kernel size	4 square pixels
Max. kernel size	19 square pixels
Number of kernels to explore for training one weak learner	100
Regularization factor	100, 500, 1,000
Max. decision tree depth	5
Shrinkage factor *γ*	0.1

### Prediction with the Kernel–Boost classifier

During the prediction phase, the classifier is applied onto the subject animal (*Drosophila*) silhouette foreground. For each pixel, the learned kernels and decision tree splitting are applied onto the image patches. Passing through all iterations gives a confidence probability that the pixel of interest belongs to a leg. From our experiments, we found that a confidence threshold of 0.6–0.65 was optimal.

### Tracking module

For tracking, the segmented leg pixels are promoted from pixel level to object level representation by grouping connected sets of identified leg pixels while rectifying irregularities and noise arising during segmentation.

By obtaining the centroid and angle-of-rotation from the silhouette image, each frame is translated and rotated such that the subject animal (*Drosophila*) is aligned with the y-axis. After a single pixel-wide skeletonization of the legs, the leg claw is identified as the endpoint at a maximum distance from the fly body. Tracking is automatically initialized by identifying the first frame with the correct number of identified legs, which are then labeled according to their geometric positions. In the case of *Myrmaplata plataleoides* (salticid spider) spider-leg tracking was manually initiated by marking each leg tip in the first frame. Subsequent tracking proceeds as normal.

Leg tracking invokes an optimal linear assignment problem in which the leg tips in the next frame have to be consistently labeled as those in the current frame, as follows.

Given tips $x_{i}^{j}$ the position of the *j*-th leg in frame *i* (in body centred coordinates), consistent labels *j* = 1, …,6 have to be assigned to ***x***_*i*+1_ of frame *i* +1.

The assignment cost is the distance between the tips across the frames and the problem seeks a global minimization of this distance to assign a label to the tips in the next frame. Formally, the problem can be stated as:

$$x_{i+1}^{\sigma (j)}=\underset{\sigma \in K(m,n)}{\text{argmin}}\sum_{j}^{m}{∥x_{i+1}^{\sigma (j)}-x_{i}^{j}∥}_{2}$$

in which there are *m* tips identified in frame *i* and *n* tips identified in frame *i* + 1 and *σ* is an injective mapping from the set of *m* elements to the set of *n* elements, subject to the constraint that ${∥x_{i+1}^{\sigma (j)}-x_{i}^{j}∥}_{2}\leq 20$, (i.e., the tip of a leg cannot move >20 pixels across a single frame).

We denote the set of all such possible mappings as *K*(*m*, *n*), using the Hungarian method [56,57] *O*(*m*^3^) combinatorial algorithm. In the event that legs are not found, the last located position of the missing legs in a previous frame is utilized to match with any leftover tips in frame *i* + 1. Similarly, in cases of leg occlusion or false negatives in identifying leg tips, the previous frame’s history is used to restore the correct identity upon leg tip reappearance. A summary of the tracking procedure and parameters is shown in Box 2. A manual correction feature in the FLLIT program allows the user to correct mislabeled legs or make adjustments to tip positions.

**Box 2. Tracking procedure**

INPUT:

  Segmented leg pixels in each frame

  Drosophila Silhouette in each frame

  Maximum Distance Constraint moved by a leg tip

 1. For *i* = 1: Total number of frames

 2. Obtain centroid position and orientation of the *Drosophila* from the binary silhouette. Translation and rotation operation to align the drosophila along the y-axis.

 3. Group segmented leg pixels as different legs and identify leg tips as the endpoints at a maximum distance from the body. This gives all leg tips ***x***_*i*_ across all frames.

 4. End for *i*

 5. Tracking initialization: identify the starting frame with all legs being visible, and label them according to geometric position. This gives $x_{start}^{j}$ for *j* = 1, …, #legs.

 6. For *i* = Starting Frame + 1: End Frame

 7. Labelling tips in frame *i*: $x_{i}^{\sigma (j)}=\underset{\sigma \in K(m,n)}{\text{argmin}}\sum_{j}^{m}{∥x_{i}^{\sigma (j)}-x_{i-1}^{j}∥}_{2}$ subject to the constraint ${∥x_{i}^{\sigma (j)}-x_{i-1}^{j}∥}_{2}$< 20 pixels with Hungarian algorithm.

 8. Recovering missing tips with last seen information and leftover tips: $x_{i}^{Missing}=\underset{\sigma \in K(\#\text{Missing},\#\text{Left}-\text{over})}{\text{argmin}}\sum_{j}^{\#Missing}{∥x_{i}^{Left-over}-x_{lastseen}^{Missing}∥}_{2}$

 9. End For

OUTPUT:

 Trajectory of leg tips from start to end frame in both the body-centered as well as arena-centered frames of reference.

## Experimental methods

### System setup and video recording

Fig 1 and S1 Fig illustrate the video setup. In brief, a Photron FastCam MC2 high speed camera was mounted below an arena containing the sample. The arena was backlit with a diffused infrared LED array. For *Drosophila*, the floor and ceiling of the arena consisted of glass microscope slides, and the arena walls were cut from 1.5-mm thick transparent acrylic sheets, representing the height of the arena. For *M*. *plataleoides* video recordings, the floor, ceiling, and walls of the arena were cut from transparent acrylic sheets. The walls of the arena were 6.5 mm high.

*Drosophila* were transferred into the arena by mouth aspiration or after brief incapacitation by cooling on ice and allowed to acclimatize for at least 5 min (mouth aspiration) or 15 min (on ice) before starting the recording. The field of view ranged from 9 mm x 9 mm square, to 12 mm x 12 mm square (10 mm x 10 mm default unless otherwise indicated). For *M*. *plataleoides*, the field of view measured 50 mm x 50 mm. Video recordings were carried out at 1,000 frames per second, with 512 x 512 pixel resolution at 25 °C, unless otherwise indicated. Videos were captured when the animal walked straight through the middle of the arena, without touching the perimeter. For efficient automated background subtraction by FLLIT, only videos where the subject animal moved at least 1.5 body lengths were used. Below this threshold, we found that the calculated background usually contained visible traces of the fly silhouette, impairing accurate extraction of the region of interest (fly body) and resulting in poor segmentation. If the user wishes to analyze videos where the subject did not traverse at least 1.5 body lengths, they should separately upload a background image (see below).

### Animal handling

*Drosophila* stocks (*Sh*^*5*^ (BL111), *Hk*^*2*^ (BL55), *Elav-Gal4* (BL8765), *ple-Gal4* (BL8848), *UAS-SNCA* (BL51376) *SCA3-flQ27* (BL33609), *SCA3-flQ84* (BL33610) and *park*^*1*^ (BL34747) were obtained from the Bloomington Drosophila Stock Centre (Indiana, USA). *Mir-263aGal4*^*KO*^*/bft*^*24*^ flies (referred to as *mir-263a*^*KO*^) were previously described [18]. Flies were reared at 25 ± 1°C in 70% relative humidity in an environmentally controlled incubator on a 12 h light–dark schedule. Crosses were set up with 20 females per bottle and flipped every 2 days to prevent overcrowding. Groups of 15–20 males were collected within 24 hrs of eclosion and aged without further CO_2_ exposure. Flies were flipped onto fresh food every 2–3 days, and vials were laid on their sides to minimize flies getting stuck in the food. For ground truth, wild-type flies were analyzed at 4–7 days. For the mutant genotypes, the age chosen for analysis was one at which approximately 50% of flies climbed below 1.5 cm (fourth etching on tubes used; see single fly climbing assay protocol below) and flies that climbed between 0.3–0.9 cm were used for recording (second and third etchings on tubes used). Based on this criteria, *yw*, *Sh*^*5*^, and *Hk*^*2*^ aged flies (Fig 4) were analyzed at 35–41 days. *Elav-Gal4>SCA3-flQ27* and *Elav-Gal4>SCA3-flQ84* flies were analyzed at 20–25 days. *Elav-Gal4>+* and *Elav-Gal4>SNCA* flies were analyzed at 48 days. *Yw* and *park*^*1*^ flies were analyzed at 35 days, *mir-263a*^*KO*^ flies were analyzed at 22–24 days. *Ple-Gal4>SCA3-flQ27* and *ple>SCA3-flQ84* flies were analyzed at 21–25 days. All data provided are from males walking upright, except for *yw*, *Sh*^*5*^, and *Hk*^*2*^ aged flies in Fig 4 and *Elav-Gal4>UAS-SCA3-flQ84* flies in Fig 4Iii, which were recorded walking upside down/inverted.

*M*. *plataleoides* were collected in Singapore and housed individually in cylindrical cages (6.5 cm x 8.5 cm) and reared to maturity in captivity. They were reared at 25 ± 1 °C and 80%–90% relative humidity, on a 12-h light–dark schedule. The spiders were fed six fruit flies (*D*. *melanogaster*) twice a week with access to water ad libitum.

### Segmentation ground truth

To empirically determine the classification threshold for leg pixel segmentation, we manually identified leg pixels in image frames randomly sampled from videos of wild-type *Drosophila*, taken using default recording parameters (8 videos, 2–3 images per video). We then determined the precision, recall, and F_0.5_ scores achieved using the FLLIT segmentation classifiers.

The blurry regions on the edges of the legs are marked with white pixels and given a weight of 0 so that they will not be important in assessing the precision and recall scores. The F_0.5_ score for the tested classifiers peaked at 0.6–0.65 (S2A Fig); hence, we selected this threshold for subsequent analyses. Users may adjust the classification threshold in the FLLIT interface based on their requirements.

### Tracking ground truth

Users were instructed to zoom in to the image frame to 800% magnification, and to label the leg-tip pixel as accurately as possible with no time constraint. One in every 20 frames (5% of each video) was annotated in this way. Different colours were used to label each leg tip, and labeled positions were then compared with leg-tip positions that were derived computationally or from another user.

#### Error identification

Misidentification errors occurred mainly during leg retraction and occlusion, and had to be corrected to prevent error propagation. Corrections of these errors were carried out to minimize the need for further corrections, while allowing for missing data. We took advantage of the algorithm’s ability to match tips only within a set distance threshold (20 pixels) across frames, by locating a correction <20 pixels away from where the leg may reappear after occlusion, and >20 pixels away from the site of misidentification, to prevent subsequent further cases of misidentification (S2C Fig). To avoid using these incorrectly annotated positions, sharp movements occurring within 1 ms were filtered out before carrying out subsequent analyses. Tips that were reported absent as a result of these corrections were included in the missing data tally.

## Background loading

Segmentation and tracking accuracy depend on clean background subtraction (the first step of image processing). As such, an automated background subtraction step was built into FLLIT. This automated background subtraction algorithm requires the subject animal to move at least a distance of 1.5 body lengths; hence, videos were made with this criteria in mind. All data shown were generated using the FLLIT-derived automated background subtraction. In most cases, this procedure performed well; in some cases, or if the subject animal does not traverse at least 1.5 body lengths, loading of a background can substantially improve segmentation and tracking (S2D Fig). A manual background can be made either by taking a separate image of the background alone or by constructing one via image processing.

### Side by side comparison to DeepLabCut [25]

#### Data set preparation

Two manually annotated *Drosophila* data sets with default settings were used as the ground truth data sets. Within each data set, 200 training images were randomly selected and the training set was prepared according to the DeepLabCut specifications. A grayscale image was converted to three image channels by repeating the grayscale image channel. Each training set consisted of 200 three-channel images together with the respective annotated tip positions of the six legs. The remaining images within the two ground truth data sets formed the testing set.

#### Training parameters

The default training hyperparameters in DeepLabCut were used. A pretrained ResNet 50 network was used as the convolutional layers for extracting image features. The Huber loss was adopted as the training loss with location refinement set to true. Training was done for 100,000 iterations separately on each training set.

#### Testing phase

The model trained on one data set was tested on both the test sets. We observed that a model trained with images from data set A will often mismatch the leg identities on the test images from data set B and vice versa. We hence manually corrected the leg identities to improve the results from DeepLabCut. These leg-tip positions labeled by DeepLabCut were then compared to the manually annotated data set to generate the deviation in tip position in pixels.

### Side by side comparison to Isakov and colleagues [22]

The above two ground truth data sets that were used for comparison to DeepLabCut were used. As the TDM method was sensitive to image contrast, each video was manually reviewed to select a section with the longest bout of straight walking whose image contrast was acceptable by the method for successful tracking. The tracking algorithm in Isakov and colleagues was then used to generate predictions for the fly centroid and tip positions of the legs. These leg-tip predictions labeled by Isakov and colleagues [22] were then compared to the manually annotated data set to generate the deviation in tip position in pixels.

### Gait parameters

The following gait parameters were analyzed: Body veering (Number of body turns normalized to the average number of strides per leg), Footprint regularity[19] (Standard deviation of the anterior extreme position, normalized to body length), Leg domain length normalized to body length, Average ratio of the hind vs mid domain length of the right and left sides, Number of pixels overlapping between leg domains, normalized to the average number of strides per leg), Stride lengths of the mid and hind legs normalized to body length, Average ratio of the hind vs mid stride lengths of the right and left sides.

### Analysis of shaking and tremor events

Shaking and tremor events were analyzed as described (Fig 4B). Matlab scripts for detecting extrema and determining tremor frequency are provided at the project website.

### Data preparation and handling

Videos were cropped so that the animal did not touch the perimeter of the arena during the trial. These videos were converted to TIFF format and analyzed with FLLIT. No data points were removed and replicates are separate animals.

### Statistical analyses

Since several distributions did not conform to normality, we used non-parametric methods for statistical analyses. For comparing two genotypes, we used the Mann-Whitney test. For comparing three or more genotypes, we used the Kruskal–Wallis test with Dunn’s multiple comparisons posthoc test. Statistical analysis was carried out using Prism 6 (GraphPad Software).

For gait signature analysis, we computed Cliff’s delta for 10 different gait parameters (Table 2). Cliff’s delta is a nonparametric measurement of effect size that reflects the likelihood that an observation from a test group is greater than an observation from a control group. It ranges from −1 (when all values in the mutant group are smaller than the control) to +1 (when all values in the mutant group are larger than the control). The greater the overlap between the two distributions, the closer to 0 Cliff’s delta will be. Unlike Cohen’s *d*, Cliff’s delta can be applied on non-normal distributions. Each mutant genotype was compared to the appropriate control as shown in S5 Fig. Cliff’s delta was calculated according to the original formulation by Norman Cliff[58] using NumPy https://www.numpy.org. 95% confidence intervals (95 CIs) were obtained via bootstrap methods[59] with 10,000 samples, using the scikits.bootstrap package: https://github.com/cgevans/scikits-bootstrap. Forest plots were created with matplotlib https://matplotlib.org.

### Body size measurement

Body length measurements were individually obtained for each fly for normalization. Three still images from the video of each fly (first, middle and last frame) were used for measurement. Using Microsoft Paint, each image frame was magnified to 800%, and the anterior-most pixel of the head and posterior-most pixel of the abdomen at the midline were labelled. The labelled images were then opened in imageJ, and the scale was input accordingly (distance in pixels: 512, known distance: varies, unit of length: mm). A line was drawn between the labelled head and abdomen tip pixels to obtain the body length. The length determined in each of the three images was then averaged to obtain the average body size.

### Single fly climbing assays

Climbing experiments were carried out between 3 and 6 pm to minimize circadian differences. Single flies were transferred to 14ml falcon tubes (Falcon #352059), with cut ends sealed with transparent plastic, and allowed to acclimate undisturbed for 15–30 min before testing. Flies were lightly tapped down to the bottom of the tube, and the climbing height attained in 30 s was measured. Tubes were then placed horizontally and retested 10–15 mins later. The average of the two technical replicates for each vial was recorded, and the height climbed for each fly was plotted as a single point. Based on the climbing assay results, all flies that climb between approximately 0.3–0.9 cm were recorded for gait analysis (Fig 3). For Fig 3D and 3E, the approximately 10 worst and approximately 10 best climbers were recorded for gait analysis.

### FLLIT software

The most recent version of FLLIT can be downloaded from https://github.com/BII-wushuang/FLLIT.

### User workflow

Users first select a data folder of image frames for analysis. They have an option to load a background, which is only necessary if the subject moves slowly or not at all through a portion of the video, as this hampers automated background subtraction. Training then automatically occurs on a subset of the data set before segmentation is carried out. The confidence threshold for classification can also be selected. Default settings are pre-loaded. Segmentation then proceeds without the need for further input. After segmentation, tracking begins. Leg claw positions are highlighted and labelled during tracking. Errors in leg identity assignment may occasionally occur when legs come in close proximity to each other, cross over, or when a leg emerges after a prolonged period of time being hidden. As leg claw positions are tracked across adjacent frames, correcting a mislabeled leg identity at the earliest point following the wrongful assignment is usually sufficient to correct leg identity in subsequent frames. The user can perform error correction either during tracking, or after tracking is completed. Tracking can then be resumed from the point of correction.

## Supporting information

S1 Fig System setup. A. Experimental setup used for video capture. Videos taken with the side view camera (that is synchronized with the top/bottom view camera) were used for occasional reference but not for tracking. B. Top view of the sample stage. Related to Fig 1.
(TIF)

S2 Fig Ground truth demonstrates the accuracy of segmentation and tracking results reported by FLLIT. A. Average F_0.5_, precision and recall scores for segmentation, at various confidence thresholds (*n* = 18 images from 8 videos). Classifier performance peaked at thresholds of 0.6–0.65. The more stringent threshold of 0.65 was selected for subsequent analyses. B. Body length measurements in pixels (anterior to wing posterior) taken under our default video recording parameters. Bars represent the means and standard deviations. C. Example of error correction for misidentification errors. i) In frame 518, leg claw R1 was misidentified to the left leg (yellow arrow) during retraction of leg R1; this error was perpetuated for multiple frames while the R1 leg was occluded. ii) A single correction was made in frame 518 (yellow arrow and circle labelled R1), >20 pixels away from the location of the misidentification. iii) After the correction (from frames 519 to 526), R1 was subsequently reported as missing, because no segmented region was found within 20 pixels of the corrected R1 position in frame 518. iv) The correctly tracked position for R1 reappears in frame 527, <20 pixels away from the correction made in frame 518. D. Effect of suboptimal automated background generation on segmentation performance. (i) The FLLIT-generated background left traces of the fly silhouette, compared to (ii) a background that was manually constructed using image processing. (iii) Poor background subtraction and segmentation as a result of (i). (iv) Improved segmentation after subtracting a manually constructed background. E. Manual annotation of the front left leg (top image; red dot within the yellow circle) by a human user, compared to segmentation failure leading to marking the left front leg claw as “absent” by the algorithm, and thus resulting in missing data (bottom image; yellow circle). F. *Drosophila* silhouette segmented using solely morphological operations (top; the red and blue pixels constitute a set of highly confident positive and negative training samples; see also Fig 1B), and the same silhouette segmented by application of a classifier after training (Bottom). G. Percentage of missing data for each video decreased after learning compared to using morphological operations alone (*P < 0*.0001 using a non-parametric Wilconxon matched-pairs signed rank test; *n* = 29 videos, 15,166 frames, 90,996 legs). H. Frequency distribution of the deviation (in pixels) between computationally and manually-derived leg claw positions when using morphological operations alone versus after application of a classifier (*n* = 106 frames, 636 leg tips from two videos). Related to Fig 2. FLLIT, Feature Learning-based LImb segmentation and Tracking.
(TIF)

S3 Fig Robustness testing of FLLIT by altering recording parameters and application of FLLIT to other arthropods. A. Representative images from videos of wild-type *Drosophila* recorded under various recording settings that were manually annotated and analyzed in (B). Default setting: default lighting/contrast, default resolution (10-mm square field of view), 1,000 fps; low contrast (decreased light intensity) versus high contrast (increased light intensity); lower resolution (12-mm field of view) versus higher resolution (9 mm); low frame rate (250 fps) versus medium frame rate (500 frames per second). B. (i) Number of leg tips not found and (ii) number of corrections required in videos of wild-type *Drosophila* monitored under different recording parameters. Default setting (*n* = 29 videos, 15,166 frames), low contrast (*n* = 9 videos, 5,678 frames), high contrast (*n* = 7 videos, 4,664 frames), lower resolution (*n* = 9 videos, 4,596 frames), higher resolution (9 mm) (*n* = 5 videos, 3,473 frames). 250 fps (*n* = 8 videos, 1,331 frames) and 500 fps (*n* = 5 videos, 1,389 frames). The graph depicts the number of corrections required per 1,000 frames, with error bars representing the means and standard deviations. (iii) Deviation (in pixels) between computationally and manually-derived leg-tip positions under the recording settings shown in (A). Data are represented as box and whiskers plots showing the 2.5 to 97.5 percentiles, with the >97.5 percentile points indicated using triangles. Settings: Default (*n* = 636 legs), low contrast (*n* = 390 legs), high contrast (*n* = 456 legs), lower resolution (*n* = 324 legs), higher resolution (*n* = 306 legs), 250 fps (*n* = 360 legs), and 500 fps (*n* = 186 legs). Bars represent the means and standard deviations. C. Representative images of the pixel resolution of *M*. *plataleoides* salticid spider leg tips, at the recording settings used in this study. Red and green insets are 10 pixels wide and show the respective boxed regions in the yellow-boxed image. D. Number of corrections required for misidentified legs, normalised to per 1,000 frames (mean = 1.2 corrections/1000 frames; *n* = 9 videos, 12,683 frames, 101,464 legs). E. Percent of missing data in each video after error correction. F. Frequency distribution of the deviation (in pixels) between computationally and manually-derived leg tip positions (*n* = 167 frames, 1,336 leg tips from 3 videos). Bars represent the means and standard deviations. See also S1 Video. FLLIT, Feature Learning-based LImb segmentation and Tracking.
(TIF)

S4 Fig Side-by-side comparison of method performance. A. Percentage missing data for ground truth videos taken under different recording parameters, when tracked with either the method from Isakov *et al*^6^ (TDM) or using FLLIT. Settings: Default (*n* = 2 videos, 730 frames), lower resolution (*n* = 2 videos, 938 frames), high contrast (*n* = 2 videos, 1,562 frames), low contrast (*n* = 2 videos, 1,322 frames). Only frames from videos where the fly walked sufficiently close to the centre of the frame could be tracked with TDM. Tracking failed for all low contrast videos. B. Frequency distribution of the deviation (in pixels) between computationally tracked and manually annotated leg-tip positions, using either TDM, FLLIT, or DLC (trained either on the same video or on a different video recorded under the same settings) (*n* = 420 legs, 2 videos; default settings). C. Deviation (in pixels) between computationally tracked and manually annotated leg-tip positions, using either TDM, FLLIT, or DeepLabCut (DLC, trained either on the same video or on a different video recorded under the same settings). Default setting (*n* = 2 videos, 420 legs), lower resolution (*n* = 2 videos, 276 legs), high contrast (*n* = 2 videos, 456 legs), low contrast (*n* = 2 videos, 390 legs). D. Percentage of frames containing at least one leg that deviated >3 pixels from the manually annotated position, when tracked using either TDM, FLLIT or DeepLabCut (DLC, trained either on the same video or on a different video recorded under the same settings). Default setting (*n* = 2 videos, 420 legs), lower resolution (*n* = 2 videos, 276 legs), high contrast (*n* = 2 videos, 456 legs), low contrast (*n* = 2 videos, 390 legs). Bars represent the means and standard deviations. FLLIT, Feature Learning-based LImb segmentation and Tracking.
(TIF)

S5 Fig Characterization of gait in fly models of SCA3 and PD. (A–D) Dot plots of the respective gait parameters shown in Fig 3. The genotypes as indicated (colored as in Fig 3A) were analyzed for the following gait parameters: (i) body veering (number of body turns normalized to the average number of strides per leg), (ii) footprint regularity (standard deviations of the anterior extreme position, normalized to body length), (iii) leg domain length normalized to body length, (iv) average ratio of the hind versus mid domain length of the right and left sides, (v) number of pixels overlapping between leg domains, normalized to the average number of strides per leg), (vi) stride lengths of the mid and hind legs normalized to body length, (vii) average ratio of the hind versus mid stride lengths of the right and left sides. *P < 0.05, **P < 0.01, ***P < 0.001, ****P < 0.0001. Genotypes examined: *Elav-Gal4>SCA3-flQ27* (*n* = 10), *Elav-Gal4>SCA3-flQ84* (*n* = 10), Elav-Gal4>+ (*n* = 9), *Elav-Gal4>SCNA* (*n* = 9), *yw* (*n* = 11), *park*^*1*^ (*n* = 10), *mir-263a*^*KO*^ (*n* = 11), *ple-Gal4>SCA3-flQ27* (*n* = 14), and *ple-Gal4>SCA3-flQ84* (*n* = 15). For panels with two genotypes (A, B, D), data were analyzed using a nonparametric Mann–Whitney test. For panels with three genotypes (C), data were analyzed using a nonparametric Kruskal–Wallis test with Dunn’s multiple comparisons posthoc test. Bars represent the means and standard deviations. Related to Fig 3. PD, Parkinsons Disease; SCA3, Spinocerebellar ataxia Type 3.
(TIF)

S1 Video Representative FLLIT-tracked video of a freely walking 4-day-old *yw* fly. FLLIT, Feature Learning-based LImb segmentation and Tracking.
(MP4)

S2 Video Representative FLLIT-tracked video of a freely walking salticid spider. FLLIT, Feature Learning-based LImb segmentation and Tracking.
(MP4)

S3 Video Representative FLLIT-tracked video of a freely walking 23-day-old *Elav-Gal4>SCA3-flQ27* fly. FLLIT, Feature Learning-based LImb segmentation and Tracking.
(MP4)

S4 Video Representative FLLIT-tracked video of a freely walking 25-day-old *Elav-Gal4>SCA3-flQ84* fly. FLLIT, Feature Learning-based LImb segmentation and Tracking.
(MP4)

S5 Video Representative FLLIT-tracked video of a freely walking 48-day-old *Elav-Gal4>SNCA* fly. FLLIT, Feature Learning-based LImb segmentation and Tracking.
(MP4)

S6 Video Representative FLLIT-tracked video of a freely walking 35-day-old *park[1]* fly. FLLIT, Feature Learning-based LImb segmentation and Tracking.
(MP4)

S7 Video Representative FLLIT-tracked video of a freely walking 22-day-old *ple-Gal4>SCA3-flQ84* fly. FLLIT, Feature Learning-based LImb segmentation and Tracking.
(MP4)

S8 Video Representative FLLIT-tracked video of a freely walking 35-day-old *yw* fly. FLLIT, Feature Learning-based LImb segmentation and Tracking.
(MP4)

S9 Video Representative FLLIT-tracked video of a freely walking 35-day-old *Hk[2]* fly. FLLIT, Feature Learning-based LImb segmentation and Tracking.
(MP4)

S1 Table Effect size summaries for Fig 3C and 3E. (PDF)

S1 Data Underlying data for all figures except Fig 3C and 3E. (XLSX)

S2 Data Underlying data for Fig 3C and 3E. (XLSX)

## Abbreviations

CSV: comma separated value
FLLIT: Feature Learning-based LImb segmentation and Tracking
fps: frames per second
PD: Parkinsons Disease
SCA3: Spinocerebellar ataxia Type 3
SNCA: alpha-synuclein
TDM: thresholding and dynamic masking methods
