# Supplementary figures and images for: Fully automated leg tracking of Drosophila neurodegeneration models reveals distinct conserved movement signatures
Source: PLoS Biol. 2019 Jun 27;17(6):e3000346. doi: 10.1371/journal.pbio.3000346 (PMC6619818; doi:10.1371/journal.pbio.3000346)

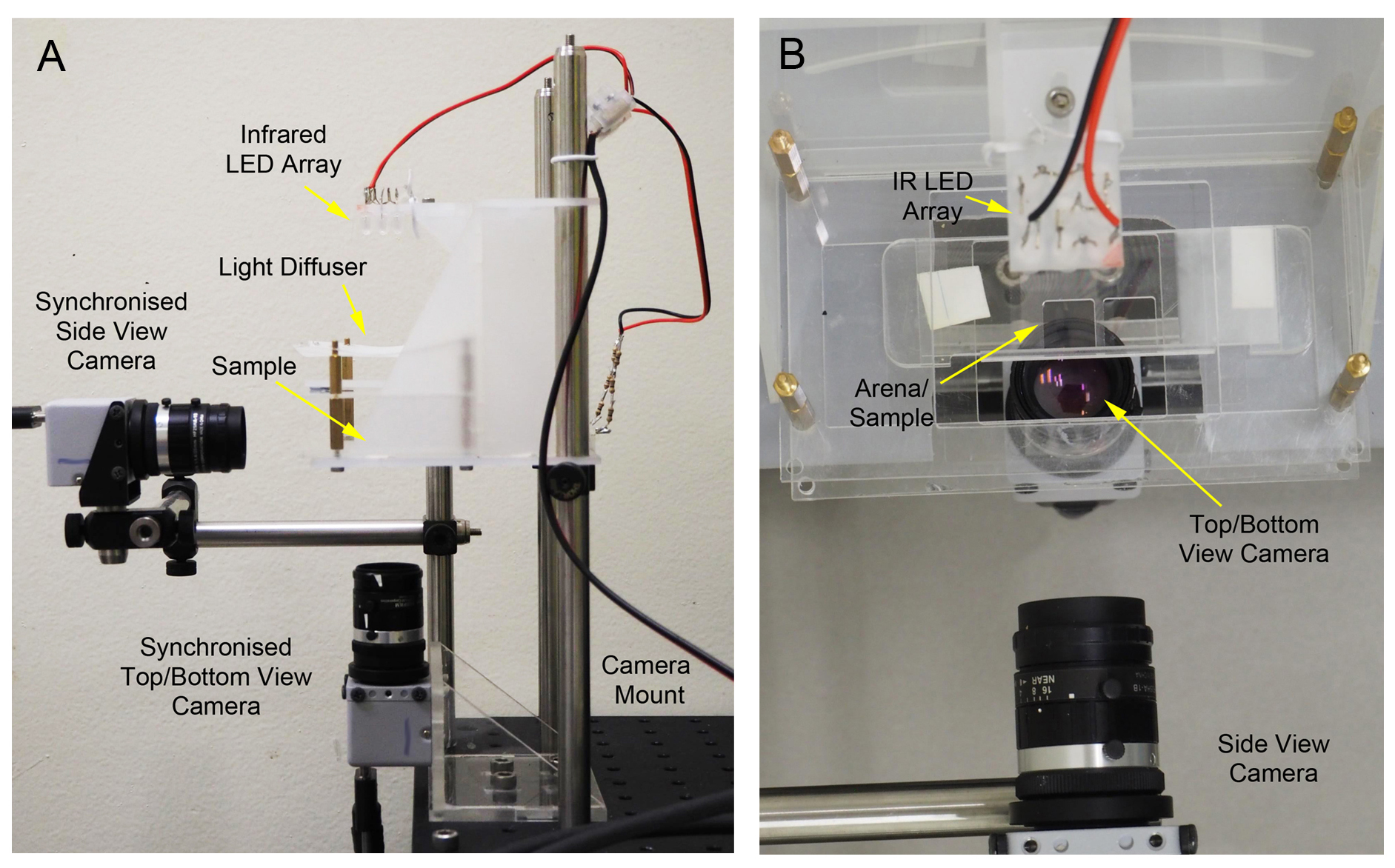

Supplement: S1 Fig — A. Experimental setup used for video capture. Videos taken with the side view camera (that is synchronized with the top/bottom view camera) were used for occasional reference but not for tracking. B. Top view of the sample stage. Related to Fig 1. (TIF) [file pbio.3000346.s001.tif]

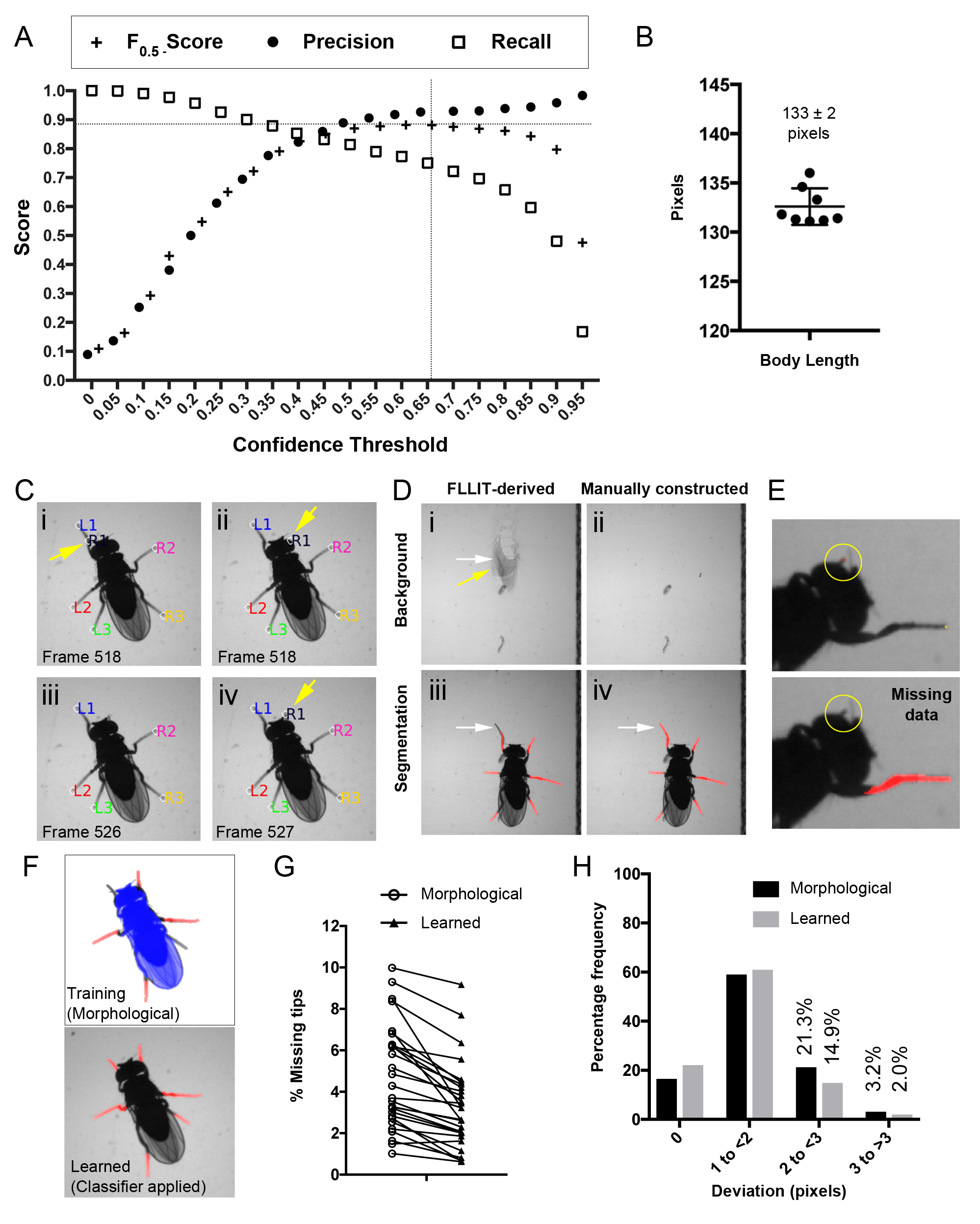

Supplement: S2 Fig — A. Average F0.5, precision and recall scores for segmentation, at various confidence thresholds (n = 18 images from 8 videos). Classifier performance peaked at thresholds of 0.6–0.65. The more stringent threshold of 0.65 was selected for subsequent analyses. B. Body length measurements in pixels (anterior to wing posterior) taken under our default video recording parameters. Bars represent the means and standard deviations. C. Example of error correction for misidentification errors. i) In frame 518, leg claw R1 was misidentified to the left leg (yellow arrow) during retraction of leg R1; this error was perpetuated for multiple frames while the R1 leg was occluded. ii) A single correction was made in frame 518 (yellow arrow and circle labelled R1), >20 pixels away from the location of the misidentification. iii) After the correction (from frames 519 to 526), R1 was subsequently reported as missing, because no segmented region was found within 20 pixels of the corrected R1 position in frame 518. iv) The correctly tracked position for R1 reappears in frame 527, <20 pixels away from the correction made in frame 518. D. Effect of suboptimal automated background generation on segmentation performance. (i) The FLLIT-generated background left traces of the fly silhouette, compared to (ii) a background that was manually constructed using image processing. (iii) Poor background subtraction and segmentation as a result of (i). (iv) Improved segmentation after subtracting a manually constructed background. E. Manual annotation of the front left leg (top image; red dot within the yellow circle) by a human user, compared to segmentation failure leading to marking the left front leg claw as “absent” by the algorithm, and thus resulting in missing data (bottom image; yellow circle). F. Drosophila silhouette segmented using solely morphological operations (top; the red and blue pixels constitute a set of highly confident positive and negative training samples; see also Fig 1B), and [file pbio.3000346.s002.tif]

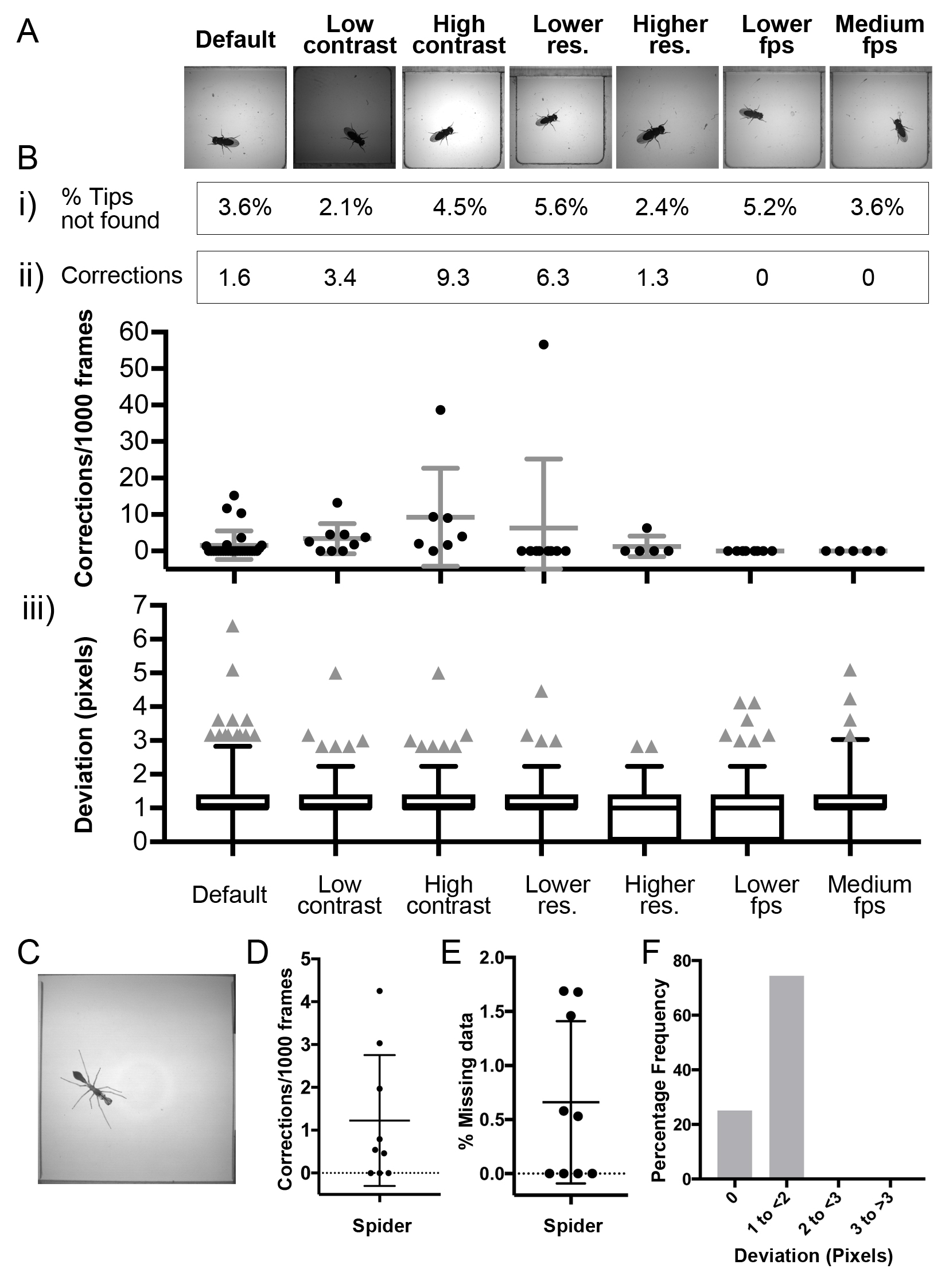

Supplement: S3 Fig — A. Representative images from videos of wild-type Drosophila recorded under various recording settings that were manually annotated and analyzed in (B). Default setting: default lighting/contrast, default resolution (10-mm square field of view), 1,000 fps; low contrast (decreased light intensity) versus high contrast (increased light intensity); lower resolution (12-mm field of view) versus higher resolution (9 mm); low frame rate (250 fps) versus medium frame rate (500 frames per second). B. (i) Number of leg tips not found and (ii) number of corrections required in videos of wild-type Drosophila monitored under different recording parameters. Default setting (n = 29 videos, 15,166 frames), low contrast (n = 9 videos, 5,678 frames), high contrast (n = 7 videos, 4,664 frames), lower resolution (n = 9 videos, 4,596 frames), higher resolution (9 mm) (n = 5 videos, 3,473 frames). 250 fps (n = 8 videos, 1,331 frames) and 500 fps (n = 5 videos, 1,389 frames). The graph depicts the number of corrections required per 1,000 frames, with error bars representing the means and standard deviations. (iii) Deviation (in pixels) between computationally and manually-derived leg-tip positions under the recording settings shown in (A). Data are represented as box and whiskers plots showing the 2.5 to 97.5 percentiles, with the >97.5 percentile points indicated using triangles. Settings: Default (n = 636 legs), low contrast (n = 390 legs), high contrast (n = 456 legs), lower resolution (n = 324 legs), higher resolution (n = 306 legs), 250 fps (n = 360 legs), and 500 fps (n = 186 legs). Bars represent the means and standard deviations. C. Representative images of the pixel resolution of M. plataleoides salticid spider leg tips, at the recording settings used in this study. Red and green insets are 10 pixels wide and show the respective boxed regions in the yellow-boxed image. D. Number of corrections required for misidentified legs, normalised to per 1,000 frames (mean = 1.2 correction [file pbio.3000346.s003.tif]

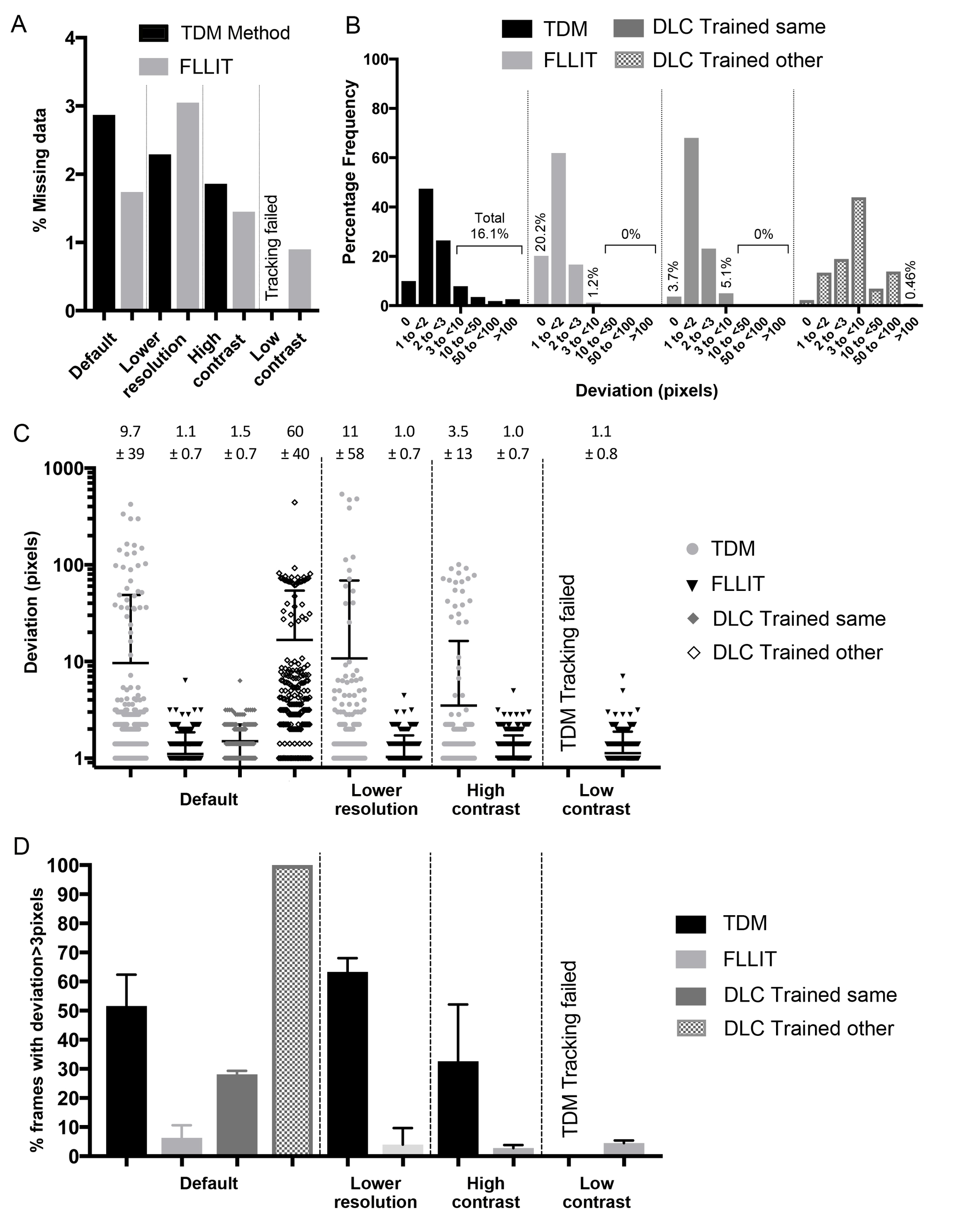

Supplement: S4 Fig — A. Percentage missing data for ground truth videos taken under different recording parameters, when tracked with either the method from Isakov et al6 (TDM) or using FLLIT. Settings: Default (n = 2 videos, 730 frames), lower resolution (n = 2 videos, 938 frames), high contrast (n = 2 videos, 1,562 frames), low contrast (n = 2 videos, 1,322 frames). Only frames from videos where the fly walked sufficiently close to the centre of the frame could be tracked with TDM. Tracking failed for all low contrast videos. B. Frequency distribution of the deviation (in pixels) between computationally tracked and manually annotated leg-tip positions, using either TDM, FLLIT, or DLC (trained either on the same video or on a different video recorded under the same settings) (n = 420 legs, 2 videos; default settings). C. Deviation (in pixels) between computationally tracked and manually annotated leg-tip positions, using either TDM, FLLIT, or DeepLabCut (DLC, trained either on the same video or on a different video recorded under the same settings). Default setting (n = 2 videos, 420 legs), lower resolution (n = 2 videos, 276 legs), high contrast (n = 2 videos, 456 legs), low contrast (n = 2 videos, 390 legs). D. Percentage of frames containing at least one leg that deviated >3 pixels from the manually annotated position, when tracked using either TDM, FLLIT or DeepLabCut (DLC, trained either on the same video or on a different video recorded under the same settings). Default setting (n = 2 videos, 420 legs), lower resolution (n = 2 videos, 276 legs), high contrast (n = 2 videos, 456 legs), low contrast (n = 2 videos, 390 legs). Bars represent the means and standard deviations. FLLIT, Feature Learning-based LImb segmentation and Tracking. (TIF) [file pbio.3000346.s004.tif]

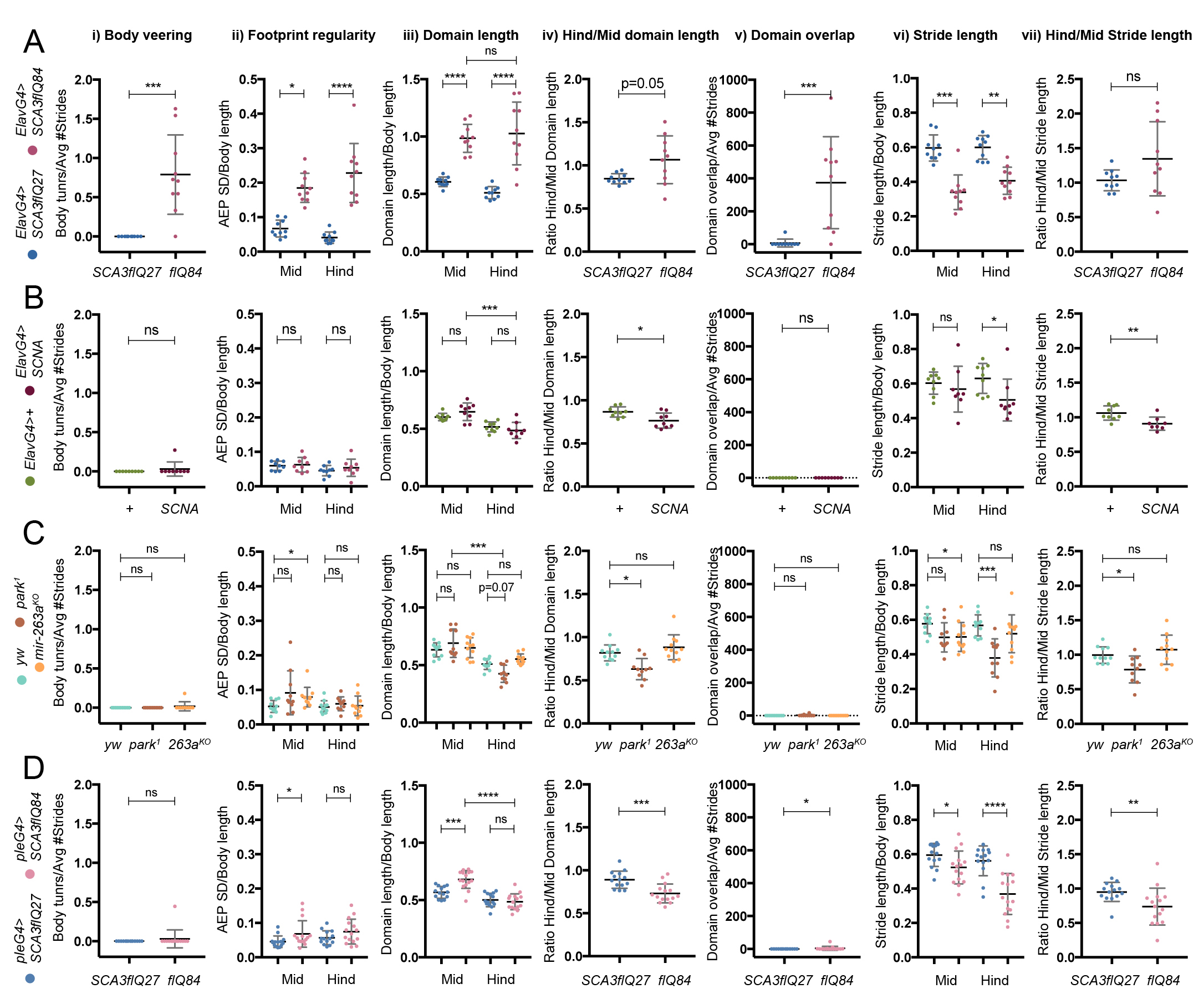

Supplement: S5 Fig — (A–D) Dot plots of the respective gait parameters shown in Fig 3. The genotypes as indicated (colored as in Fig 3A) were analyzed for the following gait parameters: (i) body veering (number of body turns normalized to the average number of strides per leg), (ii) footprint regularity (standard deviations of the anterior extreme position, normalized to body length), (iii) leg domain length normalized to body length, (iv) average ratio of the hind versus mid domain length of the right and left sides, (v) number of pixels overlapping between leg domains, normalized to the average number of strides per leg), (vi) stride lengths of the mid and hind legs normalized to body length, (vii) average ratio of the hind versus mid stride lengths of the right and left sides. *P < 0.05, **P < 0.01, ***P < 0.001, ****P < 0.0001. Genotypes examined: Elav-Gal4>SCA3-flQ27 (n = 10), Elav-Gal4>SCA3-flQ84 (n = 10), Elav-Gal4>+ (n = 9), Elav-Gal4>SCNA (n = 9), yw (n = 11), park1 (n = 10), mir-263aKO (n = 11), ple-Gal4>SCA3-flQ27 (n = 14), and ple-Gal4>SCA3-flQ84 (n = 15). For panels with two genotypes (A, B, D), data were analyzed using a nonparametric Mann–Whitney test. For panels with three genotypes (C), data were analyzed using a nonparametric Kruskal–Wallis test with Dunn’s multiple comparisons posthoc test. Bars represent the means and standard deviations. Related to Fig 3. PD, Parkinsons Disease; SCA3, Spinocerebellar ataxia Type 3. (TIF) [file pbio.3000346.s005.tif]
