# Supplementary material for: Fully automated leg tracking of Drosophila neurodegeneration models reveals distinct conserved movement signatures
Source: PLoS Biol. 2019 Jun 27;17(6):e3000346. doi: 10.1371/journal.pbio.3000346 (PMC6619818; doi:10.1371/journal.pbio.3000346)
Supplement: S1 Table — (PDF) [file pbio.3000346.s015.pdf]

Supplemental Table 1: Effect size summary
Figure 3C: Effect size summary

Gait parameter	Mutant genotype	No. of mutant flies	Control genotype	No. of control flies	Cliff's delta	95CI	Mann-Whitney P value	Kruskal Wallis	Threshold
Body veering	*Elav-Gal4>SCA3-flQ84*	10	*Elav-Gal4>SCA3-flQ27*	10	0.9	0.7; 1.0	0		p < 0.05
Footprint regularity Mid	*Elav-Gal4>SCA3-flQ84*	10	*Elav-Gal4>SCA3-flQ27*	10	1	1.0; 1.0	0		p < 0.05
Footprint regularity Hind	*Elav-Gal4>SCA3-flQ84*	10	*Elav-Gal4>SCA3-flQ27*	10	1	1.0; 1.0	0		p < 0.05
Mid domain length	*Elav-Gal4>SCA3-flQ84*	10	*Elav-Gal4>SCA3-flQ27*	10	1	1.0; 1.0	0		p < 0.05
Hind domain length	*Elav-Gal4>SCA3-flQ84*	10	*Elav-Gal4>SCA3-flQ27*	10	0.98	0.88; 1.0	0		p < 0.05
Hind vs Mid domain length	*Elav-Gal4>SCA3-flQ84*	10	*Elav-Gal4>SCA3-flQ27*	10	0.52	0.0; 0.96	0.054		NS
Domain overlap (pixels)	*Elav-Gal4>SCA3-flQ84*	10	*Elav-Gal4>SCA3-flQ27*	10	0.86	0.59; 1.0	0.001		p < 0.05
Stride length Mid legs	*Elav-Gal4>SCA3-flQ84*	10	*Elav-Gal4>SCA3-flQ27*	10	-0.9	-1.0; -0.64	0.001		p < 0.05
Stride length Hind legs	*Elav-Gal4>SCA3-flQ84*	10	*Elav-Gal4>SCA3-flQ27*	10	-0.94	-1.0; -0.76	0		p < 0.05
Hind vs Mid Stride length	*Elav-Gal4>SCA3-flQ84*	10	*Elav-Gal4>SCA3-flQ27*	10	0.4	-0.14; 0.86	0.14		NS
Body veering	*Elav-Gal4>SNCA*	9	*Elav-Gal4>+*	9	0.111	0.0; 0.333	0.374		NS
Footprint regularity Mid	*Elav-Gal4>SNCA*	9	*Elav-Gal4>+*	9	-0.012	-0.58; 0.556	1		NS
Footprint regularity Hind	*Elav-Gal4>SNCA*	9	*Elav-Gal4>+*	9	0.284	-0.259; 0.802	0.331		NS
Mid domain length	*Elav-Gal4>SNCA*	9	*Elav-Gal4>+*	9	0.481	-0.111; 0.975	0.093		NS
Hind domain length	*Elav-Gal4>SNCA*	9	*Elav-Gal4>+*	9	-0.383	-0.852; 0.185	0.185		NS
Hind vs Mid domain length	*Elav-Gal4>SNCA*	9	*Elav-Gal4>+*	9	-0.58	-0.951; -0.111	0.042		p < 0.05
Domain overlap (pixels)	*Elav-Gal4>SNCA*	9	*Elav-Gal4>+*	9	0.111	0.0; 0.333	0.374		NS
Stride length Mid legs	*Elav-Gal4>SNCA*	9	*Elav-Gal4>+*	9	-0.21	-0.778; 0.358	0.48		NS
Stride length Hind legs	*Elav-Gal4>SNCA*	9	*Elav-Gal4>+*	9	-0.704	-1.0; -0.21	0.013		p < 0.05
Hind vs Mid Stride length	*Elav-Gal4>SNCA*	9	*Elav-Gal4>+*	9	-0.802	-1.0; -0.457	0.005		p < 0.05
Body veering	*park1*	10	*yw*	11	0.1	0.0; 0.3	0.34	>0.9999	NS
Footprint regularity Mid	*park1*	10	*yw*	11	0.4	-0.109; 0.855	0.13	0.1107	NS
Footprint regularity Hind	*park1*	10	*yw*	11	0.327	-0.182; 0.8	0.218	0.5426	NS
Mid domain length	*park1*	10	*yw*	11	0.182	-0.364; 0.673	0.504	0.7937	NS
Hind domain length	*park1*	10	*yw*	11	-0.618	-0.927; -0.164	0.018	0.0758	NS
Hind vs Mid domain length	*park1*	10	*yw*	11	-0.764	-1.0; -0.382	0.003	0.0142	p < 0.05
Domain overlap (pixels)	*park1*	10	*yw*	11	0.2	0.0; 0.5	0.147	0.1257	NS
Stride length Mid legs	*park1*	10	*yw*	11	-0.564	-0.964; -0.091	0.032	0.0516	p < 0.05
Stride length Hind legs	*park1*	10	*yw*	11	-0.945	-1.0; -0.782	0	0.0002	p < 0.05
Hind vs Mid Stride length	*park1*	10	*yw*	11	-0.691	-1.0; -0.273	0.008	0.0199	p < 0.05
Body veering	*mir-263aKO*	11	*yw*	11	0.182	0.0; 0.455	0.167	0.3909	NS
Footprint regularity Mid	*mir-263aKO*	11	*yw*	11	0.719	0.355; 0.95	0.005	0.0218	p < 0.05
Footprint regularity Hind	*mir-263aKO*	11	*yw*	11	-0.041	-0.537; 0.488	0.896	>0.9999	NS
Mid domain length	*mir-263aKO*	11	*yw*	11	0.157	-0.372; 0.653	0.555	>0.9999	NS
Hind domain length	*mir-263aKO*	11	*yw*	11	0.488	0.025; 0.851	0.057	0.2035	NS
Hind vs Mid domain length	*mir-263aKO*	11	*yw*	11	0.306	-0.174; 0.752	0.237	0.6796	NS
Domain overlap (pixels)	*mir-263aKO*	11	*yw*	11	0	0.0; 0.0	1	>0.9999	NS
Stride length Mid legs	*mir-263aKO*	11	*yw*	11	-0.603	-0.934; -0.174	0.018	0.034	p < 0.05
Stride length Hind legs	*mir-263aKO*	11	*yw*	11	-0.438	-0.835; 0.025	0.088	0.2445	NS
Hind vs Mid Stride length	*mir-263aKO*	11	*yw*	11	0.107	-0.405; 0.603	0.694	>0.9999	NS
Body veering	*ple-Gal4>SCA3-flQ84*	15	*ple-Gal4>SCA3-flQ27*	14	0.067	0.0; 0.2	0.37		NS
Footprint regularity Mid	*ple-Gal4>SCA3-flQ84*	15	*ple-Gal4>SCA3-flQ27*	14	0.524	0.133; 0.867	0.017		p < 0.05
Footprint regularity Hind	*ple-Gal4>SCA3-flQ84*	15	*ple-Gal4>SCA3-flQ27*	14	0.267	-0.171; 0.657	0.23		NS
Mid domain length	*ple-Gal4>SCA3-flQ84*	15	*ple-Gal4>SCA3-flQ27*	14	0.762	0.448; 0.99	0.001		p < 0.05
Hind domain length	*ple-Gal4>SCA3-flQ84*	15	*ple-Gal4>SCA3-flQ27*	14	-0.219	-0.629; 0.219	0.326		NS
Hind vs Mid domain length	*ple-Gal4>SCA3-flQ84*	15	*ple-Gal4>SCA3-flQ27*	14	-0.714	-0.943; -0.39	0.001		p < 0.05
Domain overlap (pixels)	*ple-Gal4>SCA3-flQ84*	15	*ple-Gal4>SCA3-flQ27*	14	0.333	0.133; 0.6	0.022		p < 0.05
Stride length Mid legs	*ple-Gal4>SCA3-flQ84*	15	*ple-Gal4>SCA3-flQ27*	14	-0.486	-0.848; -0.076	0.028		p < 0.05
Stride length Hind legs	*ple-Gal4>SCA3-flQ84*	15	*ple-Gal4>SCA3-flQ27*	14	-0.8	-1.0; -0.524	0		p < 0.05
Hind vs Mid Stride length	*ple-Gal4>SCA3-flQ84*	15	*ple-Gal4>SCA3-flQ27*	14	-0.629	-0.933; -0.257	0.004		p < 0.05

Figure 3E: Effect size summary

Gait parameter	Mutant genotype	No. of mutant flies	Control genotype	No. of control flies	Cliff's delta	95CI	Mann-Whitney P value	Mann-Whitney Threshold
Body veering	ElavGal4>UAS-SCA3-flQ84 poor climbers	9	UAS-SCA3-flQ84>+	10	0.889	0.667; 1.0	0	p < 0.05
Footprint regularity Mid	ElavGal4>UAS-SCA3-flQ84 poor climbers	9	UAS-SCA3-flQ84>+	10	1	1.0; 1.0	0	p < 0.05
Footprint regularity Hind	ElavGal4>UAS-SCA3-flQ84 poor climbers	9	UAS-SCA3-flQ84>+	10	0.822	0.4; 1.0	0.003	p < 0.05
Mid domain length	ElavGal4>UAS-SCA3-flQ84 poor climbers	9	UAS-SCA3-flQ84>+	10	0.911	0.667; 1.0	0.001	p < 0.05
Hind domain length	ElavGal4>UAS-SCA3-flQ84 poor climbers	9	UAS-SCA3-flQ84>+	10	0.822	0.4; 1.0	0.003	p < 0.05
Hind vs Mid domain length	ElavGal4>UAS-SCA3-flQ84 poor climbers	9	UAS-SCA3-flQ84>+	10	0.156	-0.422; 0.689	0.596	NS
Domain overlap (pixels)	ElavGal4>UAS-SCA3-flQ84 poor climbers	9	UAS-SCA3-flQ84>+	10	0.889	0.667; 1.0	0	p < 0.05
Stride length Mid legs	ElavGal4>UAS-SCA3-flQ84 poor climbers	9	UAS-SCA3-flQ84>+	10	-0.489	-1.0; 0.067	0.079	NS
Stride length Hind legs	ElavGal4>UAS-SCA3-flQ84 poor climbers	9	UAS-SCA3-flQ84>+	10	-0.889	-1.0; -0.644	0.001	p < 0.05
Hind vs Mid Stride length	ElavGal4>UAS-SCA3-flQ84 poor climbers	9	UAS-SCA3-flQ84>+	10	-0.111	-0.689; 0.489	0.713	NS
Body veering	ElavGal4>UAS-SCA3-flQ84 good climbers	11	UAS-SCA3-flQ84>+	10	0.727	0.455; 1.0	0.001	p < 0.05
Footprint regularity Mid	ElavGal4>UAS-SCA3-flQ84 good climbers	11	UAS-SCA3-flQ84>+	10	0.891	0.655; 1.0	0.001	p < 0.05
Footprint regularity Hind	ElavGal4>UAS-SCA3-flQ84 good climbers	11	UAS-SCA3-flQ84>+	10	0.964	0.836; 1.0	0	p < 0.05
Mid domain length	ElavGal4>UAS-SCA3-flQ84 good climbers	11	UAS-SCA3-flQ84>+	10	0.927	0.745; 1.0	0	p < 0.05
Hind domain length	ElavGal4>UAS-SCA3-flQ84 good climbers	11	UAS-SCA3-flQ84>+	10	0.818	0.491; 1.0	0.002	p < 0.05
Hind vs Mid domain length	ElavGal4>UAS-SCA3-flQ84 good climbers	11	UAS-SCA3-flQ84>+	10	0.236	-0.291; 0.727	0.379	NS
Domain overlap (pixels)	ElavGal4>UAS-SCA3-flQ84 good climbers	11	UAS-SCA3-flQ84>+	10	1	1.0; 1.0	0	p < 0.05
Stride length Mid legs	ElavGal4>UAS-SCA3-flQ84 good climbers	11	UAS-SCA3-flQ84>+	10	-0.164	-0.655; 0.364	0.549	NS
Stride length Hind legs	ElavGal4>UAS-SCA3-flQ84 good climbers	11	UAS-SCA3-flQ84>+	10	-0.273	-0.727; 0.255	0.307	NS
Hind vs Mid Stride length	ElavGal4>UAS-SCA3-flQ84 good climbers	11	UAS-SCA3-flQ84>+	10	-0.255	-0.745; 0.255	0.342	NS
Body veering	ElavGal4>UAS-SNCA poor climbers	11	UAS-SNCA>+	11	0	0.0; 0.0	11	NS
Footprint regularity Mid	ElavGal4>UAS-SNCA poor climbers	11	UAS-SNCA>+	11	0.058	-0.438; 0.57	0.844	NS
Footprint regularity Hind	ElavGal4>UAS-SNCA poor climbers	11	UAS-SNCA>+	11	0.24	-0.256; 0.719	0.358	NS
Mid domain length	ElavGal4>UAS-SNCA poor climbers	11	UAS-SNCA>+	11	-0.421	-0.851; 0.074	0.101	NS
Hind domain length	ElavGal4>UAS-SNCA poor climbers	11	UAS-SNCA>+	11	-0.719	-1.0; -0.306	0.005	p < 0.05
Hind vs Mid domain length	ElavGal4>UAS-SNCA poor climbers	11	UAS-SNCA>+	11	-0.554	-0.901; -0.124	0.03	p < 0.05
Domain overlap (pixels)	ElavGal4>UAS-SNCA poor climbers	11	UAS-SNCA>+	11	0	0.0; 0.0	11	NS
Stride length Mid legs	ElavGal4>UAS-SNCA poor climbers	11	UAS-SNCA>+	11	-0.603	-0.917; -0.19	0.018	p < 0.05
Stride length Hind legs	ElavGal4>UAS-SNCA poor climbers	11	UAS-SNCA>+	11	-0.752	-0.967; -0.405	0.003	p < 0.05
Hind vs Mid Stride length	ElavGal4>UAS-SNCA poor climbers	11	UAS-SNCA>+	11	-0.587	-0.901; -0.157	0.022	p < 0.05
Body veering	ElavGal4>UAS-SNCA good climbers	12	UAS-SNCA>+	11	0	0.0; 0.0	11	NS
Footprint regularity Mid	ElavGal4>UAS-SNCA good climbers	12	UAS-SNCA>+	11	-0.242	-0.697; 0.242	0.34	NS
Footprint regularity Hind	ElavGal4>UAS-SNCA good climbers	12	UAS-SNCA>+	11	0.258	-0.227; 0.712	0.31	NS
Mid domain length	ElavGal4>UAS-SNCA good climbers	12	UAS-SNCA>+	11	-0.455	-0.833; 0.0	0.069	NS
Hind domain length	ElavGal4>UAS-SNCA good climbers	12	UAS-SNCA>+	11	-0.576	-0.924; -0.152	0.021	p < 0.05
Hind vs Mid domain length	ElavGal4>UAS-SNCA good climbers	12	UAS-SNCA>+	11	-0.091	-0.576; 0.409	0.735	NS
Domain overlap (pixels)	ElavGal4>UAS-SNCA good climbers	12	UAS-SNCA>+	11	0.083	0.0; 0.25	0.384	NS
Stride length Mid legs	ElavGal4>UAS-SNCA good climbers	12	UAS-SNCA>+	11	-0.515	-0.879; -0.076	0.039	p < 0.05
Stride length Hind legs	ElavGal4>UAS-SNCA good climbers	12	UAS-SNCA>+	11	-0.591	-0.909; -0.182	0.018	p < 0.05
Hind vs Mid Stride length	ElavGal4>UAS-SNCA good climbers	12	UAS-SNCA>+	11	-0.318	-0.773; 0.197	0.207	NS
